# Rapid Detection of Mobilized Colistin Resistance using a Nucleic Acid Based Lab-on-a-Chip Diagnostic System

**DOI:** 10.1038/s41598-020-64612-1

**Published:** 2020-05-21

**Authors:** Jesus Rodriguez-Manzano, Nicolas Moser, Kenny Malpartida-Cardenas, Ahmad Moniri, Lenka Fisarova, Ivana Pennisi, Adhiratha Boonyasiri, Elita Jauneikaite, Alireza Abdolrasouli, Jonathan A. Otter, Frances Bolt, Frances Davies, Xavier Didelot, Alison Holmes, Pantelis Georgiou

**Affiliations:** 1 NIHR Health Protection Research Unit in Healthcare Associated Infections and Antimicrobial Resistance, Department of Infectious Disease, Faculty of Medicine, Imperial College London, London, United Kingdom; 2 Centre for Bio-Inspired Technology, Department of Electrical and Electronic Engineering, Faculty of Engineering, Imperial College London, London, United Kingdom; 3 Department of Infectious Disease Epidemiology, School of Public Health, Imperial College London, London, United Kingdom; 4 Imperial College Healthcare NHS Trust, St Mary’s Hospital, London, United Kingdom; 5 School of Life Sciences and Department of Statistics, University of Warwick, Coventry, United Kingdom

**Keywords:** Lab-on-a-chip, Microbiology techniques, Infectious diseases, Diagnosis

## Abstract

The increasing prevalence of antimicrobial resistance is a serious threat to global public health. One of the most concerning trends is the rapid spread of Carbapenemase-Producing Organisms (CPO), where colistin has become the last-resort antibiotic treatment. The emergence of colistin resistance, including the spread of mobilized colistin resistance (*mcr*) genes, raises the possibility of untreatable bacterial infections and motivates the development of improved diagnostics for the detection of colistin-resistant organisms. This work demonstrates a rapid response for detecting the most recently reported *mcr* gene, *mcr*−9, using a portable and affordable lab-on-a-chip (LoC) platform, offering a promising alternative to conventional laboratory-based instruments such as real-time PCR (qPCR). The platform combines semiconductor technology, for non-optical real-time DNA sensing, with a smartphone application for data acquisition, visualization and cloud connectivity. This technology is enabled by using loop-mediated isothermal amplification (LAMP) as the chemistry for targeted DNA detection, by virtue of its high sensitivity, specificity, yield, and manageable temperature requirements. Here, we have developed the first LAMP assay for *mcr*−9 - showing high sensitivity (down to 100 genomic copies/reaction) and high specificity (no cross-reactivity with other *mcr* variants). This assay is demonstrated through supporting a hospital investigation where we analyzed nucleic acids extracted from 128 carbapenemase-producing bacteria isolated from clinical and screening samples and found that 41 carried *mcr*−9 (validated using whole genome sequencing). Average positive detection times were 6.58 ± 0.42 min when performing the experiments on a conventional qPCR instrument (*n* = 41). For validating the translation of the LAMP assay onto a LoC platform, a subset of the samples were tested (*n* = 20), showing average detection times of 6.83 ± 0.92 min for positive isolates (*n* = 14). All experiments detected *mcr*−9 in under 10 min, and both platforms showed no statistically significant difference (*p*-value > 0.05). When sample preparation and throughput capabilities are integrated within this LoC platform, the adoption of this technology for the rapid detection and surveillance of antimicrobial resistance genes will decrease the turnaround time for DNA detection and resistotyping, improving diagnostic capabilities, patient outcomes, and the management of infectious diseases.

## Introduction

Antimicrobial resistance is a serious threat to global healthcare systems which poses a challenge for modern medicine and compromises effective infectious disease management. Of particular concern is the rapid spread of Carbapenemase-Producing Organisms (CPO)^1–3^ which are associated with high morbidity and mortality rates^4^. Carbapenemases are *β*-lactamases (*bla*) that are resistant to the carbapenems, a class of highly effective antibiotic agents. Therefore, therapeutic options are severely restricted with clinical management often relying upon “last line” antibiotics, primarily colistin. The emergence of colistin resistance through acquired mobilized colistin resistance (*mcr*) genes or chromosomal mutations is therefore a significant clinical and public health concern, raising the possibility of untreatable bacterial infections. In 2016, the first *mcr* variant, *mcr*−1, was reported in a clinical bacterial isolate^5^. Since then, several other *mcr* variants (*mcr*−1 to *mcr*−8) have been identified in over 40 countries across five different continents^6^. Very recently, the new variant *mcr*−9 has been reported and detected in a multidrug-resistant *Salmonella enterica* from a patient in Washington State^7^. Antimicrobial susceptibility testing for colistin, such as the broth microdilution methods recommended by CLSI and EUCAST guidelines, is challenging in diagnostic laboratories as current methodologies are limited by specificity/sensitivity issues and turnaround time^8^. The prevalence of colistin resistance is therefore likely to be under-reported, poorly characterized and outbreaks may go undetected.

Several diagnostic tests have been developed for the detection of colistin resistance^9^. Phenotypic assays, mostly culture-based methods, are generally recommended for under-resourced laboratories, whereas molecular tests, such as microarrays and polymerase chain reaction (PCR), are suggested for well-resourced ones^9^. Unfortunately, none of these methods combine all the ideal features for simple, efficient, economical and fast turnaround time^10^, limiting their widespread deployment. Among molecular assays, loop-mediated isothermal amplification (LAMP) is emerging as a popular nucleic acid amplification method due to its simplified thermal management, high sensitivity and high specificity^11,12^. Detection of *mcr*−1 to *mcr*−5 genes by LAMP has been demonstrated previously^13^. Moreover, other isothermal chemistries such as recombinase polymerase amplification have also been applied to *mcr*−1^14^. However, these chemistries conventionally rely on fluorescent detection of the amplicon produced^15^, requiring complex and costly hardware, often restricting it to specialized laboratories. Alternative solutions based on colorimetric detection, enabled by microfluidics and cell-phone technology, provide promising point-of-care approaches, however, compromise dynamic range of quantification^16–18^.

Electrochemical-based DNA sensors offer high sensitivity, selectivity, large scale integration, detection on miniaturized hardware, fast response and low cost^19^. Ion-sensitive field-effect transistors (ISFETs) are emerging potentiometric sensors for nucleic-acid based applications. Implementation in unmodified complementary metal-oxide-semiconductor (CMOS) technology provides ISFETs with inherent sensitivity to pH, which has enabled DNA detection by employing an adapted version of LAMP, called pH-LAMP, that allows changes in pH to occur during nucleic acid amplification^20–22^. Recently, in Malpartida-Cardenas *et al*.^23^, it was shown that this approach holds significant potential for the development of a cheap, portable and quantitative diagnostic tool. This breakthrough was demonstrated in a lab-based environment using an external thermal controller in conjunction with a desktop computer. Moreover, recent work from our lab has demonstrated a fully portable Lab-on-a-Chip (LoC) platform which has integrated thermal management within the diagnostic platform and uses a smartphone application (AndroidOS) for data acquisition, visualization and cloud connectivity^24–26^.

**Figure 1:**
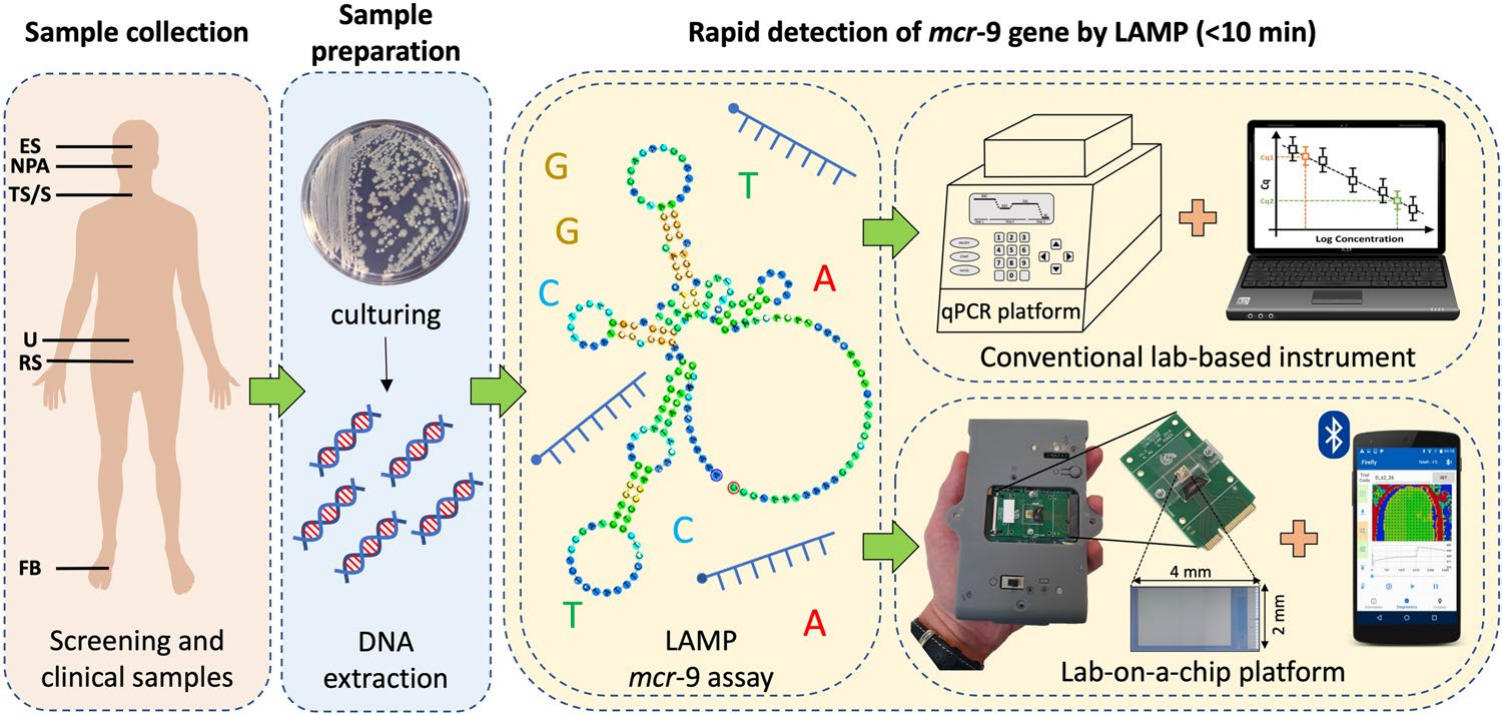
Diagnostic workflow for detection of *mcr*−9 using LAMP. Clinical and screening samples are collected from: ES, eye swab; NPA, nasopharyngeal aspirate; TS, throat swab; S, sputum; U, urine; RS, rectal swab; and FB, foot biopsy. Subsequently, samples are cultured and nucleic acids are extracted in a microbiology lab. Following this, rapid detection of *mcr*−9 is performed using our new LAMP assay on a conventional lab-based instrument connected to a desktop computer and a state-of-the-art lab-on-a-chip platform linked to a smartphone.

In this work, we explore the first application of LAMP for the rapid detection of *mcr*−9 in a conventional quantitative PCR instrument and the aforementioned LoC platform, referred to as qLAMP and eLAMP, respectively. The experimental workflow is depicted in Fig. 1 and summarized as follows: (1) through supporting a hospital investigation, 128 CPO-positive clinical and screening samples have been collected; (2) pure bacteria were cultured and nucleic acids extracted; (3) developed and characterized a new LAMP assay for detecting *mcr*−9 in under 10 minutes using a qPCR instrument; and (4) translated the new LAMP assay onto an ISFET-based LoC platform. We envision that the approach described in this work, when integrated with a sample preparation module and multiplexing capabilities, can be extended to other drug resistances, and its implementation in health care facilities will greatly expand diagnostic capabilities, improving patient outcomes and infection prevention & control.

## Results

### LAMP assay for *mcr*−9 gene detection

**Figure 2:**
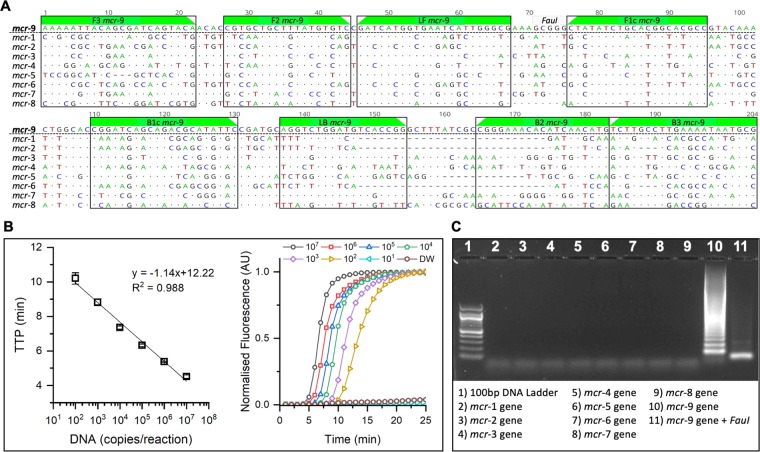
Sequence alignment, primers location and *mcr*−9 LAMP assay performance. (**A**) Sequence alignment for *mcr*−1 to *mcr*−9 showing priming region and the location of the *FauI* restriction site. Compared to the reference sequence, *mcr*−9, mismatches in the alignment are displayed as AGTC. Matched nucleotides are represented as dots and gaps are represented as dashes. (**B**) Standard curve and real-time amplification profiles obtained with the *mcr*−9 LAMP assay and synthetic DNA at concentrations ranging from 10^2^ to 10^7^ copies per reaction (including non-template control). Experiments were carried-out in a real-time qPCR platform in triplicates. (**C**) Gel electrophoresis confirming specificity of *mcr*−9 LAMP assay. Line 1 shows a 100 bp DNA ladder. Lines 2 to 10 show the amplification product obtained from synthetic DNA carrying *mcr*−1 to *mcr*−9 sequences against the *mcr*−9 LAMP assay. Line 11 shows the specific *mcr*−9 LAMP product after digestion with *FauI*.

Based on the nucleotide sequence alignment of *mcr* gene variants, specific LAMP primers for the *mcr*−9 gene detection were designed. The LAMP assay has an amplicon size of 204 bp and consists of six primers targeting eight different regions: two outer primers (F3/B3), two inner primers (FIP/BIP) and two loop primers (LF/LB). Primer sequences are provided in Table S1. A sequence alignment for *mcr*−1 to *mcr*−9 indicating the location of the *mcr*−9 LAMP primers is shown in Fig. 2A. It can be observed that many single nucleotide polymorphisms exist across the selected region and BlastN analysis of primer sequences against the NCBI database showed 100% homology and 100% coverage for the *mcr*−9 variant genes only. Using synthetic DNA containing the *mcr*−9 gene, the LAMP assay showed an excellent lower limit of detection of 100 copies/ reaction with a good correlation curve between DNA copies per reaction and time-to-positive (TTP) values, and standard curve was linear from 10^7^ to 10^2^ (*R*^2^ = 0.988) corresponding to a TTP ranging from 4 to 10 min, respectively (Fig. 2B). To evaluate the specific amplification of the LAMP assay, synthetic DNA containing *mcr*−1 to *mcr*−9 sequences were tested as templates at a concentration of 10^5^ copies/reaction. After the LAMP reaction, the amplification products were subjected to agarose gel electrophoresis. The *mcr*−9 synthetic DNA showed a positive LAMP pattern and the rest of DNA samples were negative (Fig. 2C). In addition, specificity of the LAMP-positive product was confirmed by digestion of the amplicon by *FauI* restriction enzyme digestion (sequence shown in Fig. 2A). Results presented in Fig. 2 indicate that the *mcr*−9 LAMP assay is highly sensitive and specific.

### Detecting *mcr*−9 in clinical bacterial isolates using qLAMP

To look for putative clinical isolates harboring this colistin resistance *mcr*−9 gene, the LAMP assay was applied to extracted genomic DNA samples from 128 CPO isolates (see Table 1). All isolates were collected between 2012–2019 from clinical or screening samples routinely processed by the Microbiology Department at Charing Cross Hospital, Imperial College Healthcare NHS Trust. The pure bacterial strains were identified using matrix-assisted laser desorption/ionization time-of-flight mass spectrometry (MALDI-TOF MS) as *Acinetobacter* sp. (*n* = 5), *Citrobacter* sp. (*n* = 7), *Enterobacter* spp. (*n* = 33), *Escherichia* sp. (*n* = 14), *Klebsiella* spp. (*n* = 61), *Proteus* sp. (*n* = 1), *Pseudomonas* sp. (*n* = 4), *Raoultella* sp. (*n* = 1) and *Serratia* sp. (*n* = 2). The carbapenemase-producing mechanisms were confirmed using commercially available diagnostic tests based on qPCR and immunochromatography. Carbapenemases were determined as a single enzyme in 121 strains (*bla*_GES−5_ = 2; *bla*_IMP_ = 53; *bla*_KPC_ = 8; *bla*_NDM_ = 39; *bla*_OXA−23_ = 1; *bla*_OXA−48_ = 13; *bla*_OXA−58_ = 1; *bla*_VIM_ = 4) and as a combination in seven isolates (*bla*_IMP_ + *bla*_OXA−48_ = 1; *bla*_IMP_ + *bla*_OXA−58_ = 1; *bla*_IMP_ + *bla*_NDM_ + *bla*_OXA−51−like_ = 1; *bla*_NDM_ + *bla*_OXA−48_ = 3; *bla*_NDM_ + *bla*_OXA−51_ = 1).

**Table 1:** Clinical carbapenemase-producing organisms analysed by the *mcr*−9 LAMP assay.

Species (MALDI-TOF MS)	Carbapenemase	Positive *mcr*-9 Isolates/Analysed Samples
*Acinetobacter* sp.	*bla*_IMP_ and *bla*_OXA−58_	0/1
	*bla*_IMP_, *bla*_NDM_ and *bla*_OXA−51-like_	0/1
	*bla*_OXA−23_	0/1
	*bla*_OXA−51_ and *bla*_NDM_	0/1
	*bla*_OXA−58_	0/1
*Citrobacter* sp.	*bla*_IMP_	1/1
	*bla*_KPC_	0/2
	*bla*_OXA−48_	1/3
	*bla*_VIM_	0/1
*Enterobacter* spp.	*bla*_IMP_	23/31
	*bla*_IMP_ and *bla*_OXA−48_	1/1
	*bla*_VIM_	0/1
*Escherichia coli*	*bla*_IMP_	3/3
	*bla*_NDM_	0/5
	*bla*_NDM_ and *bla*_OXA−48_	0/1
	*bla*_OXA−48_	0/5
*Klebsiella* spp.	*bla*_GES−5_	0/2
	*bla*_IMP_	10/15
	*bla*_KPC_	0/5
	*bla*_NDM_	0/33
	*bla*_NDM_ and *bla*_OXA−48_	0/2
	*bla*_OXA−48_	0/4
*Proteus mirabilis*	*bla*_NDM_	0/1
*Pseudomonas aeruginosa*	*bla*_IMP_	1/2
	*bla*_VIM_	0/2
*Raoultella planticola*	*bla*_IMP_	1/1
*Serratia marcescens*	*bla*_KPC_	0/1
	*bla*_OXA−48_	0/1

All isolates were negative for *mcr*−1 to 8 by qPCR and whole genome sequencing. A detailed description of each isolate, including bacterial species, date of sampling, specimen type, its origin, antibiotic resistance mechanisms, TTP obtained by the *mcr*−9 LAMP, and WGS results can be found in Tables S2 and S3.

**Figure 3:**
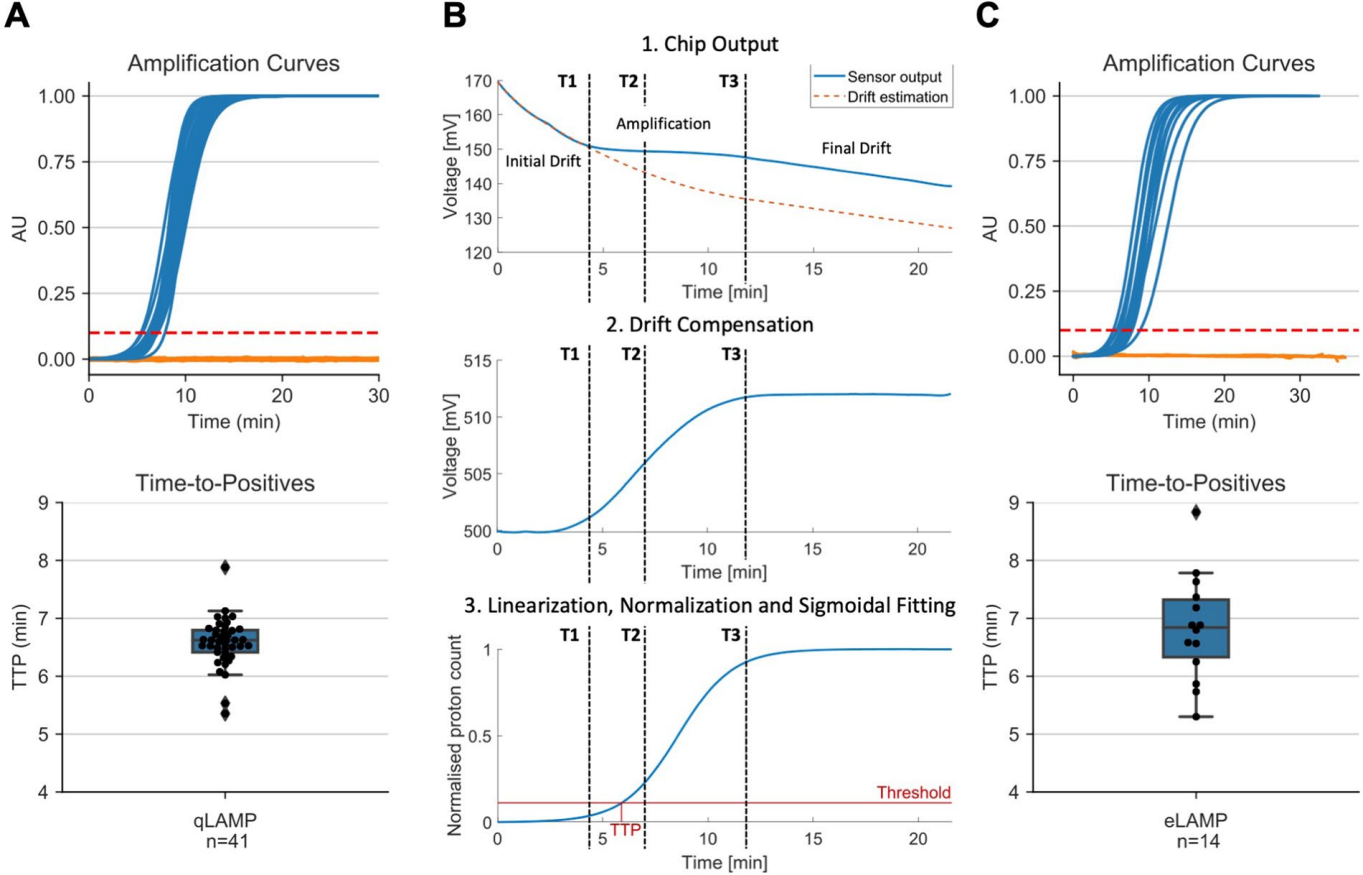
Performance of *mcr*−9 LAMP assay using qLAMP and eLAMP. (**A**) Output from qLAMP. Top panel shows the normalized negative (n = 87) and positive (n = 41) amplification curves in orange and blue, respectively. Bottom panel shows the distribution of qLAMP TTP for positive isolates. (**B**) Algorithm to extract amplification curve and TTP from LoC ISFET array output. (**C**) Output from eLAMP. Top panel shows the normalized negative (n = 6) and positive (n = 14) amplification curves in orange and blue, respectively. Bottom panel shows the distribution of eLAMP TTP for positive isolates. TTP was extracted using a threshold at 10% of the maximum. See Figures S1–S4 and Table S4 for detailed information.

The performed LAMP assay was positive for 41 samples. Interestingly, from these *mcr*−9 positive samples, 39 were carrying *bla*_IMP_, one was *bla*_IMP_ + *bla*_OXA−48_ and another one was carrying *bla*_OXA−48_. This suggests the horizontal transfer of a plasmid encoding both *mcr*−9 and *bla*_IMP_, which could result in resistance to the carbapenems and reduced susceptibility to colistin, limiting therapeutic options. However, this analysis is outside the scope of this study. The *mcr* gene was present in *Citrobacter* sp., *Enterobacter* sp., *Escherichia* sp., *Klebsiella* sp. and *Pseudomonas* sp., and all positive isolates were collected between 2016–2019. To validate these findings, we performed whole genome sequencing (WGS) analysis on 56 bacterial isolates, including all *mcr*−9 encoding isolates and 15 negative controls. The WGS data showed 100% concordance with the LAMP results. All samples were negative for *mcr*−1 to *mcr*−8 by qPCR and WGS. As shown in Fig. 3A, the TTP for all positive samples were under 10 minutes with an average value of 6.58 ± 0.42 minutes. The results presented in this section demonstrate that the developed *mcr*−9 LAMP assay allows for rapid, sensitive and specific detection of this emerging colistin resistant gene, making this assay a promising starting point for the development of a near-patient screening test.

### Translating the assay into eLAMP

In order to translate the LAMP assay onto the pH-based Lab-on-a-Chip platform, we use an adapted version of LAMP, called pH-LAMP, that allows changes in pH to occur during nucleic acid amplification (see Materials and Methods). The output of the on-chip amplification (measured in mV) consists of 4368 time-series (associated to each individual sensor) which are proportional to the pH change during the reaction (plus inherent sensor drift). The algorithm for processing the data from the ISFET array is as follows, and illustrated in Fig. 3B. Firstly, the outputs from all exposed sensors are averaged to yield the raw output curve. To extract the signal, the algorithm finds the inflection point of the curve (T2), i.e. when the rate of pH change is at its maximum. Subsequently, three reaction regions are then identified using the following method:

1. 0–1: no amplification; the output is only drift.
2. T1–T3: amplification is occurring; the output is the sum of drift and pH signal.
3. T3–end: amplification products have saturated; the output is only drift.

The drift is compensated by using an exponential approximation with the data points in regions 1 and 3. Finally, the output is fitted using a 4-parameter sigmoid and normalized such that a threshold can be applied to extract a time-to-positive, similar to that of qLAMP.

To validate our in-house platform, twenty of the isolates were analyzed, including 14 out of the 41 *mcr*−9 LAMP positive isolates. As shown in Fig. 3C, the TTP for all positive samples were also under 10 minutes with an average value of 6.83 ± 0.92 minutes. The mean between the TTP distributions obtained from qLAMP and eLAMP was found not to be statistically significant (*p* = 0.34) - indicating both instruments provide comparable results.

## Discussion

To effectively address the threats of emerging multi-drug resistant pathogens, efforts are needed to use state-of-the-art technologies in order to provide a rapid response to prevent the misuse of antibiotics. In this paper, we investigate the use of loop-mediated isothermal amplification to detect the most recently identified variant of mobilized colistin resistance in carbapenemase-producing organisms (i.e. multi-drug resistances). With current available antibiotics, bacteria carrying carbapenem- and colistin- resistance, become potentially untreatable and represents a global threat.

To date, there have been no reports on the detection of *mcr*−9 using any isothermal amplification chemistry. In this study, we first develop a novel LAMP assay for *mcr*−9 using a recently reported sequence^7^. The proposed assay was evaluated in a conventional lab-based instrument through supporting a hospital investigation where 128 carbapenemase-producing organisms were tested. We found 41 positive for *mcr*−9 across multiple bacterial species - demonstrating the ubiquitous presence of this colistin resistant gene. Furthermore, we used a subset of the positive samples, to investigate the use of a cutting-edge Lab-on-a-Chip platform which integrates semiconductor technology, avoiding bulky and complex hardware, coupled with our LAMP assay, removing the need for sophisticated thermal management. Results show that both instruments are capable of detecting *mcr*−9 in under 10 minutes.

Although the results in this manuscript present a milestone towards providing a rapid diagnostic solution at the bedside, there are several aspects that need to be further addressed. Firstly, due to a wide range of sample types in this study, the sample preparation (including culturing and nucleic acid extraction) is not integrated within the LoC platform, thereby presenting a bottleneck on the turnaround time (currently 8–24 hours). Several rapid sample preparation methods are commercially available to extract nucleic acids directly from samples, such as QuickExtract DNA Extraction Solution from Epicenter (3–8 min), however coupling them with the LoC platform is outside the scope of this manuscript. Secondly, samples were analyzed individually as only a single chamber was mounted on top of the chip, limiting the throughput of experiments. More importantly, the single chamber limits the number of targets per sample, such as detecting other *mcr* or carbapenem-resistant genes. There are multiple avenues for increasing the number of targets per experiment, such as: (i) Microfluidic solutions (e.g. SlipChip) which offer an affordable mechanism for generating multiple chambers^27–30^; or (ii) data-driven approaches which have recently been shown to perform single-channel multiplexing in a single chamber for carbapenemase-producing organisms using only real-time amplification data^31–33^. Finally, it is important to further investigate the relationship between carrying the *mcr*−9 gene and its phenotypic implications, i.e. being resistant to colistin.

The results presented in this work suggest that this approach could provide a versatile solution for the rapid detection of colistin and other antimicrobial resistances, supporting clinical management, surveillance and antimicrobial stewardship. Future work will consider combining this work with a sample preparation module and high-throughput mechanisms, providing a fully integrated platform to rapidly respond to emerging global threats outside the laboratory.

## Materials and Methods

### Samples

In this study, the *mcr*−9 LAMP assay was designed using synthetic DNA, and bacterial strains were used to validate its performance.

(i) Synthetic DNA. Double-stranded synthetic DNA (gBlock Gene fragments) containing *mcr*−1 to *mcr*−9 gene sequences (ranging from 1,617 to 1,898 bp) were purchased from Life Technologies (ThermoFisher Scientific) and resuspended in Tris-EDTA buffer to 10 ng/μL stock solutions (stored at −20 °C until further use). The concentrations of all DNA stock solutions were determined using a Qubit 3.0 fluorimeter (Life Technologies). Sequences are provided in Table S5.
(ii) Bacterial strains and culture conditions. A total of 128 non-duplicated *Enterobacteriaceae* isolates were collected between 2012–2019 from clinical or screening samples routinely processed by the hospital Microbiology Department at Imperial College Healthcare NHS Trust (Ethics protocol 06/Q0406/20). All isolates were identified as carbapenemase producers. Species identification was performed using MALDI-TOF MS and carbapenemase mechanisms were determined using the Xpert Carba-R (Cepheid) or Resist-3 O.K.N assay (Corisbio). The *bla*_GES−5_ and *Acinetobacter* species were further characterized at the national reference laboratory, Public Health England. The isolates were subcultured on appropriate growth media and incubated at 37 °C overnight, and the genomic DNA was extracted using GenElute Bacterial Genomic DNA kit (Sigma-Aldrich) following the manufacturer’s instructions.

### LAMP primer design

A set of LAMP primers specific to the *mcr*−9 gene was designed based on the sequences reported by Carroll *et al*.^7^ (GenBank: MK791138.1 and NG_064792.1) using the Primer Explorer software, version 5.0 (http://primerexplorer.jp/lampv5e/index.html). Thirty-nine other sequences including variants of *mcr*−1 to *mcr*−8 strains were retrieved from NCBI’s GenBank database (https://www.ncbi.nlm.nih.gov/genbank/) to analyze primer specificity *in silico*. GenBank accession numbers can be found in Table S6. All sequences were aligned using the MUSCLE algorithm^34^ in Geneious 10.0.5 software^35^. All primers used in this study were purchased from Life Technologies (ThermoFisher Scientific).

### LAMP reaction conditions

The amplification reaction conditions are as follows:

(i) qLAMP. Each mix contained: 1.5 μL of 10× isothermal buffer (New England BioLabs), 0.9 μL of MgSO_4_ (100mM stock), 2.1 μL of dNTPs (10 mM stock), 0.375 μL of BSA (20 mg/μL stock), 2.4 μL of Betaine (5 M stock), 0.375 μL of SYTO 9 Green (20 μM stock), 0.6 μL of Bst 2.0 DNA polymerase (8000 U/μL stock), 3 μL of different concentrations of synthetic DNA or gDNA, 1.5 μL of 10× LAMP primer mixture (20 μL of BIP/FIP, 10 μM of LF/LB, and 2.5 μM B3/F3) and enough nuclease-free water (ThermoFisher Scientific) to bring the volume to 15 μL. Reactions were performed at 63 °C for 20 min. One melting cycle was performed at 0.1 °C/s from 65 °C up to 97 °C for validation of the specificity of the products. Experiments were carried out in triplicates (using 5 μL for each reaction) loading the reactions into 96-well plates and using a LightCycler 96 Real-Time PCR System (LC96) (Roche Diagnostics).
(ii) eLAMP. Each mix contained: 3.0 μL of 10× isothermal pH-based buffer (pH 8.5–9), 1.8 μL of MgSO_4_ (100mM stock), 1.68 μL of dNTPs (25 mM stock), 1.8 μL of BSA (20mg/μL stock), 4.8 μL of Betaine (5M stock), 0.15 μL of Bst 2.0 DNA polymerase (120000 U/μL stock), 3 μL of different concentrations of synthetic DNA or gDNA, 0.75 μL of NaOH (0.2M stock), 3.0 μL of 10× LAMP primer mixture and enough nuclease- free water (ThermoFisher Scientific) to bring the volume to 30 μL. Reactions were performed at 63 °C for 20 min. Reactions of 10 μL each were loaded into a disposable cartridge and experiments were carried-out using our in-house platform.

### Limit of detection for *mcr*−9 LAMP assay

Analytical sensitivity was evaluated with 10-fold dilutions of synthetic DNA containing the *mcr*−9 sequence, ranging from 10^2^ to 10^7^ copies per reaction. Each experimental condition was run in triplicate. The LAMP assays were performed in a LightCycler 96 and the data was analyzed using LC96 System software version SW1.1.

### Cross-reactivity of *mcr*−9 LAMP assay

The specificity of the assay was validated through multiple methods as follows:

(i) Restriction digestion. The specificity of *mcr*−9 amplification was determined by the restriction digestion of the LAMP products using the *FauI* enzyme (New England BioLabs). The restriction was performed for 1 h at 55 °C, using the CutSmart buffer provided by the manufacturer. The digested LAMP products were subjected to electrophoresis on a 1.5% agarose gel at 120 V for 1 h and then visualized under UV light, using the BioSpectrum Imaging System (Ultra-Violet Products Ltd.), after staining with SYBR Safe DNA Gel Stain (InvitroGen). Experiments were performed using *mcr*−1 to *mcr*−9 synthetic DNA.
(ii) PCR analysis. The presence of *mcr*−1 to *mcr*−8 was tested by using published primer sets and reaction conditions^36^.
(iii) Whole genome sequencing. Bacterial isolates positive for the *mcr*−9 LAMP assay were further analyzed by whole genome sequencing. Multiplexed Illumina Nextera XT-generated libraries were sequenced on the NextSeq. 500 platform (Illumina) using 2× 150-bp paired-end mode with a target average coverage of 100-fold. Read quality was assessed using FastQC v0.11.6^37^. Raw reads were assembled using SPAdes v3.11^38^ and annotated with Prokka v.1.12^39^. Antimicrobial resistance genes, including *mcr*−9 were identified using the Basic Local Alignment Search Tool (BLAST) searching against the databases of ResFinder v3.1.0^40^ and the Comprehensive Antibiotic Research Database^41^ with a threshold of 98% identity and 60% query match length.

### Lab-on-a-chip Platform

The LoC system (depicted in Fig. S5) is comprised of^23–26^: (1) Diagnostic platform powered by a rechargeable battery containing: a Peltier heating module with an embedded PID controller to enable on-board isothermal nucleic acid amplification; digital algorithms for data acquisition; and a Bluetooth module to transmit information to a smartphone. (2) Single-use cartridge consisting of a CMOS ISFET microchip (4,368 sensors, 2 × 4 *mm*) for DNA sensing and a microfluidic chamber made of acrylic to contain the amplification reaction. To guarantee proper sealing, biocompatible double-sided tape is used to attach the manifold to the chip and PCR tape is used to cover the chamber during the amplification reaction. A reference electrode (0.03 mm Ag/AgCl) is placed between the chip and the double-sided tape such that it is in contact with the chemical solution, and then soldered on the PCB track to allow electrical contact with the motherboard. The diagnostic platform and single-use cartridge is shown in Fig. 1. (3) Smartphone application (developed for AndroidOS) to interface with the chip via Bluetooth in order to gather the diagnostic data and perform geotagging of the diagnostic location through the phone GPS. The application supports optional storage of the data on a cloud server (Amazon web services).

### Statistical analysis

Time-to-positive data is presented as mean TTP ± standard deviation; *p*-values were calculated by Welch’s unequal variance t-test in Python (v3.6) SciPy Package (v1.4.1). A *p*-value equal to 0.05 was considered as a threshold for statistical significance. To estimate the number of required samples for translating the LAMP assay onto the LoC platform we used the following formula^42^:

1 $$n\ge \frac{{1.96}^{2}p\mathrm{(1}-p)}{{x}^{2}}$$

where *p* is the suspected sensitivity and x is the desired margin of error. We define the true-positive rate (sensitivity) as the proportion of *mcr*−9 positive which are correctly identified by the lab-on-a-chip platform compared to the gold standard qPCR instrument. We suspected the sensitivity and specificity to be 95% with a desired margin of error of 10%. Under these conditions, the number of required samples is 18.2 (rounded up to 19) therefore we tested a total 20 samples (14 positive and 6 negative).

## Supplementary information

Supplementary information.
