# Supplementary material for: Rapid Detection of Mobilized Colistin Resistance using a Nucleic Acid Based Lab-on-a-Chip Diagnostic System
Source: Sci Rep. 2020 May 21;10:8448. doi: 10.1038/s41598-020-64612-1 (PMC7242339; doi:10.1038/s41598-020-64612-1)

Supporting Information

Rapid Detection of Mobilized Colistin Resistance using a Nucleic Acid Based Lab-on-a-Chip Diagnostic System

Jesus Rodriguez-Manzano[1,2,*], Nicolas Moser[2], Kenny Malpartida-Cardenas[2], Ahmad Moniri[2], Lenka Fisarova[2], Ivana Pennisi[2], Adhiratha Boonyasiri[1], Elita Jauneikaite[1,3], Alireza Abdolrasouli[1], Jonathan A. Otter[1,4], Frances Bolt[1], Frances Davies[1], Xavier Didelot[5], Alison Holmes[1] & Pantelis Georgiou[2]

[1]*NIHR Health Protection Research Unit in Healthcare Associated Infections and Antimicrobial Resistance, Department of Infectious Disease, Faculty of Medicine, Imperial College London, London, United Kingdom.*

[2]*Centre for Bio-Inspired Technology, Department of Electrical and Electronic Engineering, Faculty of Engineering, Imperial College London, London, United Kingdom.*

[3]*Department of Infectious Disease Epidemiology, School of Public Health, Imperial College London, London, United Kingdom.*

[4]*Imperial College Healthcare NHS Trust, St Mary's Hospital, London, United Kingdom.*

[5]*School of Life Sciences and Department of Statistics, University of Warwick, Coventry, United Kingdom.*

*Corresponding Author: j.rodriguez-manzano@imperial.ac.uk

Supporting information includes:

- sequences of the *mcr*-9 LAMP primers (**Table S1**)
- results and information of the carbapenem-resistant clinical isolates used in this study (**Table S2** and **S3**),
- summary of results obtained from samples analysed on-chip (**Table S4**).
- nucleotide sequences for synthetic double-stranded DNA (**Table S5**),
- accession number list for alignments (**Table S6**),
- raw and processed on-chip data (**Figures S1, S2, S3 and S4**)
- annotated photograph of the LoC platform without the case (**Figure S5**)

Table S1. Sequences of LAMP primers for specific detection of the *mcr*-9 gene.

**Primer ID**	**Sequence (5'-3')**
F3_mcr9	AAAAATTACAGCGATCAGTACA
B3_mcr9	CGCATTATTTTCAAGGCAAGA
FIP_mcr9	GGCGTGCCGTGCAGATATAG-CGTGCTGCTTTATGTGTC
BIP_mcr9	CGGATCAGCAGACGCATATTC-CATGTTGATGTGTTTCCCG
LF_mcr9	CGCCCAATGATTCACCATGATC
LB_mcr9	AGGTCTGGATGTCACCGG

F3, forward outer primer; **B3**, backward outer primer; **FIP**, forward inner primer; **BIP**, backward inner primer; **LF**, loop forward primer; **LB**, loop backward primer.

Table S2. Carbapenem-resistant isolates analyzed by whole genome sequencing.

Isolate ID	Bacterial strains (MALDI-TOF)	Collected	Specimen Type	Source	Carbapenemase	mcr9 gene	
						qLAMP (TTP)	WGS
#1	*Acinetobacter baumannii*	16/05/2019	S	RS	IMP1, NDM & OXA-51 like	NEG	NEG
#2	*Acinetobacter baumannii*	27/05/2019	S	RS	IMP1 and OXA58	NEG	NEG
#3	*Citrobacter freundii*	21/02/2016	S	RS	OXA48	POS (6.62 min)	POS
#4	*Citrobacter freundii*	19/08/2017	S	RS	IMP1	POS (6.47 min)	POS
#5	*Enterobacter bugandensis*	12/06/2017	S	RS	IMP1	NEG	NEG
#6	*Enterobacter cloacae*	14/06/2016	S	RS	IMP1	NEG	NEG
#7	*Enterobacter cloacae*	16/06/2016	S	RS	OXA48 and IMP1	POS (5.35 min)	POS
#8	*Enterobacter cloacae*	28/08/2017	S	RS	IMP1	NEG	NEG
#9	*Enterobacter cloacae*	03/10/2017	S	RS	IMP1	NEG	NEG
#10	*Enterobacter cloacae*	11/10/2017	S	RS	IMP1	POS (6.53 min)	POS
#11	*Enterobacter cloacae*	20/11/2017	S	TS	IMP1	NEG	NEG
#12	*Enterobacter cloacae*	28/01/2018	S	RS	IMP1	POS (7 min)	POS
#13	*Enterobacter cloacae*	06/02/2018	S	RS	IMP1	POS (6.86 min)	POS
#14	*Enterobacter cloacae*	06/02/2018	S	RS	IMP1	POS (6.81 min)	POS
#15	*Enterobacter cloacae*	21/02/2018	S	RS	IMP1	POS (6.63 min)	POS
#16	*Enterobacter cloacae*	22/04/2018	S	RS	IMP1	POS (6.63 min)	POS
#17	*Enterobacter cloacae*	26/06/2018	S	RS	IMP1	POS (6.24 min)	POS
#18	*Enterobacter cloacae*	06/08/2018	S	RS	IMP1	POS (6.41 min)	POS
#19	*Enterobacter cloacae*	18/08/2018	S	RS	IMP1	POS (6.73 min)	POS
#20	*Enterobacter cloacae*	06/09/2018	S	RS	IMP1	POS (6.8 min)	POS
#21	*Enterobacter cloacae*	12/01/2019	S	RS	IMP1	POS (6.55 min)	POS
#22	*Enterobacter cloacae*	08/02/2019	S	RS	IMP1	NEG	NEG
#23	*Enterobacter cloacae*	13/03/2019	S	RS	IMP1	NEG	NEG
#24	*Enterobacter cloacae*	25/03/2019	S	RS	IMP1	POS (6.52 min)	POS
#25	*Enterobacter cloacae*	21/02/2018	S	RS	IMP1	POS (7.03 min)	POS
#26	*Enterobacter hormaechei*	15/12/2016	S	RS	IMP1	POS (7.03 min)	POS
#27	*Enterobacter hormaechei*	26/01/2017	S	RS	IMP1	POS (6.32 min)	POS
#28	*Enterobacter hormaechei*	26/07/2017	S	RS	IMP1	POS (6.67 min)	POS
#29	*Enterobacter hormaechei*	22/01/2018	S	RS	IMP1	POS (6.5 min)	POS
#30	*Enterobacter sp.*	09/08/2017	S	RS	IMP1	POS (6.08 min)	POS
#31	*Enterobacter sp.*	03/10/2017	CL	U	IMP1	POS (6.78 min)	POS
#32	*Enterobacter sp.*	05/10/2017	S	RS	IMP1	POS (6.49 min)	POS
#33	*Enterobacter sp.*	08/05/2019	CL	NPA	IMP1	POS (6.39 min)	POS
#34	*Enterobacter sp.*	08/05/2019	CL	S	IMP1	NEG	NEG
#35	*Enterobacter xiangfangensis*	13/03/2019	S	RS	IMP1	POS (7.88 min)	POS
#36	*Enterobacter xiangfangensis*	27/05/2019	S	RS	IMP1	POS (6.83 min)	POS
#37	*Escherichia coli*	08/02/2018	S	RS	IMP1	POS (6.6 min)	POS
#38	*Escherichia coli*	25/04/2019	S	RS	IMP1	POS (6.21 min)	POS
#39	*Escherichia coli*	11/05/2019	CL	U	IMP1	POS (6.27 min)	POS
#40	*Klebsiella aerogenes*	07/01/2019	S	RS	IMP1	NEG	NEG
#41	*Klebsiella aerogenes*	27/02/2019	S	RS	IMP1	POS (6.34 min)	POS
#42	*Klebsiella aerogenes*	08/04/2019	S	RS	IMP1	POS (6.71 min)	POS
#43	*Klebsiella oxytoca*	08/04/2019	S	RS	IMP1	NEG	NEG
#44	*Klebsiella pneumoniae*	17/07/2017	S	RS	IMP1	NEG	NEG
#45	*Klebsiella pneumoniae*	23/07/2017	S	RS	IMP1	POS (5.53 min)	POS
#46	*Klebsiella pneumoniae*	09/09/2017	S	RS	IMP1	POS (6.02 min)	POS
#47	*Klebsiella pneumoniae*	19/09/2017	S	RS	IMP1	NEG	NEG
#48	*Klebsiella pneumoniae*	28/10/2017	S	RS	IMP1	POS (6.63 min)	POS
#49	*Klebsiella pneumoniae*	28/01/2018	S	RS	IMP1	NEG	NEG
#50	*Klebsiella pneumoniae*	02/04/2018	S	RS	IMP1	POS (6.91 min)	POS
#51	*Klebsiella pneumoniae*	09/04/2018	S	RS	IMP1	POS (6.93 min)	POS
#52	*Klebsiella pneumoniae*	07/09/2018	S	RS	IMP1	POS (6.52 min)	POS
#53	*Klebsiella pneumoniae*	10/09/2018	S	RS	IMP1	POS (6.79 min)	POS
#54	*Klebsiella quasipneumoniae*	11/02/2019	S	RS	IMP1	POS (6.63 min)	POS
#55	*Pseudomonas aeruginosa*	20/11/2018	S	RS	IMP1	POS (7.13 min)	POS
#56	*Raoultella planticola*	02/10/2017	CL	FB	IMP1	POS (6.53 min)	POS

MALDI-TOF MS, matrix-assisted laser desorption/ionization time-of-flight mass spectrometry**; qLAMP,** quantitative loop-mediated isothermal amplification; **TTP,** time-to-positive; **WGS,** whole genome sequencing; **S,** screen; **CL,** clinical; **RS,** rectal swab; **FB,** foot biopsy; **U,** urine; **TS,** throat swab; **NPA,** nasopharyngeal aspirate; **S,** sputum; **ES,** eye swab. All samples were negative for *mcr*-1 to *mcr*-8 by PCR.

Table S3. Remaining isolates analyzed by the *mcr*-9 LAMP assay.

Isolate ID	Bacterial strains (MALDI-TOF)	Collected	Source	Carbapenemase	*mcr*-9 qLAMP
#57	*Acinetobacter baumannii*	05/01/2016	Rectal Swab	OXA5 and NDM	NEG
#58	*Acinetobacter baumannii*	26/02/2015	BAL	OXA23	NEG
#59	*Acinetobacter* sp.	08/09/2015	Perineum swab	OXA58	NEG
#60	*Citrobacter freundii*	06/01/2016	Rectal Swab	OXA48	NEG
#61	*Citrobacter freundii*	13/12/2015	Rectal Swab	OXA48	NEG
#62	*Citrobacter freundii*	02/04/2016	Rectal Swab	VIM	NEG
#63	*Citrobacter* sp.	01/10/2017	Rectal Swab	KPC	NEG
#64	*Citrobacter* sp.	15/10/2017	Rectal Swab	KPC	NEG
#65	*Enterobacter cloacae*	14/01/2015	Bone (Tibia R)	VIM	NEG
#66	*Escherichia coli*	01/08/2016	Rectum	NDM	NEG
#67	*Escherichia coli*	05/03/2015	High vaginal swab	NDM	NEG
#68	*Escherichia coli*	14/03/2014	Wound Swab (Penis Prepuce)	OXA48	NEG
#69	*Escherichia coli*	20/10/2017	Rectal Swab	OXA48	NEG
#70	*Escherichia coli*	04/04/2015	MSU	OXA48	NEG
#71	*Escherichia coli*	25/11/2012	Wound Swab (Foot R)	OXA48	NEG
#72	*Escherichia coli*	21/07/2013	Blood	OXA48	NEG
#73	*Escherichia coli*	09/04/2015	Perineum swab	NDM	NEG
#74	*Escherichia coli*	01/12/2014	Rectal Swab	NDM and OXA48	NEG
#75	*Escherichia coli*	23/11/2015	Faeces	NDM	NEG
#76	*Escherichia coli*	26/12/2015	Rectal Swab	NDM	NEG
#77	*Klebsiella oxytoca*	13/08/2015	Urine	GES5	NEG
#78	*Klebsiella oxytoca*	26/09/2015	Rectal Swab	GES5	NEG
#79	*Klebsiella pneumoniae*	08/10/2012	Wound Swab	KPC	NEG
#80	*Klebsiella pneumoniae*	22/03/2014	MSU	KPC	NEG
#81	*Klebsiella pneumoniae*	10/09/2017	Rectal Swab	KPC	NEG
#82	*Klebsiella pneumoniae*	08/04/2015	MSU	KPC	NEG
#83	*Klebsiella pneumoniae*	11/09/2017	Rectal Swab	KPC	NEG
#84	*Klebsiella pneumoniae*	25/12/2015	MSU	NDM	NEG
#85	*Klebsiella pneumoniae*	18/12/2015	Rectal Swab	NDM	NEG
#86	*Klebsiella pneumoniae*	02/08/2016	Throat swab	NDM	NEG
#87	*Klebsiella pneumoniae*	18/04/2015	Rectal Swab	NDM	NEG
#88	*Klebsiella pneumoniae*	23/04/2015	Urine	NDM	NEG
#89	*Klebsiella pneumoniae*	25/04/2015	Rectal Swab	NDM	NEG
#90	*Klebsiella pneumoniae*	04/12/2016	Rectal Swab	NDM	NEG
#91	*Klebsiella pneumoniae*	29/01/2015	Mouth Swab	NDM	NEG
#92	*Klebsiella pneumoniae*	20/04/2015	Rectal Swab	NDM	NEG
#93	*Klebsiella pneumoniae*	10/05/2015	Perineum Swab	NDM	NEG
#94	*Klebsiella pneumoniae*	07/01/2016	Rectal Swab	OXA48	NEG
#95	*Klebsiella pneumoniae*	02/07/2014	MSU	OXA48	NEG
#96	*Klebsiella pneumoniae*	22/01/2015	Drain Fluid Abdomen	OXA48	NEG
#97	*Klebsiella pneumoniae*	28/12/2015	Nose/axilla/groin	NDM and OXA48	NEG
#98	*Klebsiella pneumoniae*	17/02/2015	Wound Swab (Leg R)	NDM and OXA48	NEG
#99	*Klebsiella pneumoniae*	09/12/2014	Perineum swab	NDM	NEG
#100	*Klebsiella pneumoniae*	20/04/2015	Rectal Swab	NDM	NEG
#101	*Klebsiella pneumoniae*	15/04/2015	Rectal Swab	NDM	NEG
#102	*Klebsiella pneumoniae*	07/05/2015	Perineum Swab	NDM	NEG
#103	*Klebsiella pneumoniae*	23/03/2018	Sputum	NDM	NEG
#104	*Klebsiella pneumoniae*	29/04/2015	Rectal Swab	NDM	NEG
#105	*Klebsiella pneumoniae*	02/07/2014	Catheter Urine	NDM	NEG
#106	*Klebsiella pneumoniae*	08/03/2015	Clean catch urine	NDM	NEG
#107	*Klebsiella pneumoniae*	10/04/2015	Urine	NDM	NEG
#108	*Klebsiella pneumoniae*	19/04/2015	Perineum Swab	NDM	NEG
#109	*Klebsiella pneumoniae*	17/05/2015	Rectal Swab	NDM	NEG
#110	*Klebsiella pneumoniae*	03/10/2015	Rectal Swab	NDM	NEG
#111	*Klebsiella pneumoniae*	16/08/2015	Rectal Swab	NDM	NEG
#112	*Klebsiella pneumoniae*	19/04/2015	Wound Swab (Groin)	NDM	NEG
#113	*Klebsiella pneumoniae*	16/09/2015	Rectal Swab	NDM	NEG
#114	*Klebsiella pneumoniae*	07/06/2015	Rectal Swab	NDM	NEG
#115	*Klebsiella pneumoniae*	07/07/2015	Rectal Swab	NDM	NEG
#116	*Klebsiella pneumoniae*	03/06/2015	Rectal Swab	NDM	NEG
#117	*Klebsiella pneumoniae*	20/05/2015	Rectal Swab	NDM	NEG
#118	*Klebsiella pneumoniae*	29/07/2015	RT Foot Tissue	NDM	NEG
#119	*Klebsiella pneumoniae*	10/08/2015	Rectal Swab	NDM	NEG
#120	*Klebsiella pneumoniae*	12/07/2015	Perineum Swab	NDM	NEG
#121	*Klebsiella pneumoniae*	21/07/2015	Rectal Swab	NDM	NEG
#122	*Klebsiella* sp.	15/12/2015	Faeces	OXA48	NEG
#123	*Proteus mirabilis*	07/02/2014	Catheter Urine	NDM	NEG
#124	*Pseudomonas aeruginosa*	25/03/2015	Wound Swab Flank R	VIM	NEG
#125	*Pseudomonas aeruginosa*	01/11/2013	Sputum	VIM	NEG
#126	*Pseudomonas aeruginosa*	31/03/2015	Throat swab	IMP	NEG
#127	*Serratia marcescens*	10/10/2017	Rectal Swab	KPC	NEG
#128	*Serratia marcescens*	28/01/2015	Bone (Tibia L)	OXA48	NEG

All samples were negative for *mcr*-1 to *mcr*-8 by PCR.

Table S4. Bacterial isolated analyzed by LoC platform.

Isolate ID	Bacterial strains	Collected	Carbapenemase	*mcr*-9 gene	
				eLAMP (TTP)	WGS
#04	*Citrobacter freundii*	19/08/2017	IMP1	POS (6.80 min)	POS
#05	*Enterobacter bugandensis*	12/06/2017	IMP1	NEG	NEG
#10	*Enterobacter cloacae*	11/10/2017	IMP1	POS (7.18 min)	POS
#11	*Enterobacter cloacae*	20/11/2017	IMP1	NEG	NEG
#13	*Enterobacter cloacae*	06/02/2018	IMP1	POS (7.63 min)	POS
#14	*Enterobacter cloacae*	06/02/2018	IMP1	POS (5.73 min)	POS
#23	*Enterobacter cloacae*	13/03/2019	IMP1	NEG	NEG
#24	*Enterobacter cloacae*	25/03/2019	IMP1	POS (6.57 min)	POS
#26	*Enterobacter hormaechei*	15/12/2016	IMP1	POS (7.37 min)	POS
#28	*Enterobacter hormaechei*	26/07/2017	IMP1	POS (8.83 min)	POS
#34	*Enterobacter sp.*	08/05/2019	IMP1	NEG	NEG
#37	*Escherichia coli*	08/02/2018	IMP1	POS (6.88 min)	POS
#41	*Klebsiella aerogenes*	27/02/2019	IMP1	POS (6.88 min)	POS
#45	*Klebsiella pneumoniae*	23/07/2017	IMP1	POS (6.25 min)	POS
#46	*Klebsiella pneumoniae*	09/09/2017	IMP1	POS (6.58 min)	POS
#47	*Klebsiella pneumoniae*	19/09/2017	IMP1	NEG	NEG
#49	*Klebsiella pneumoniae*	28/01/2018	IMP1	NEG	NEG
#51	*Klebsiella pneumoniae*	09/04/2018	IMP1	POS (5.30 min)	POS
#53	*Klebsiella pneumoniae*	10/09/2018	IMP1	POS (5.87 min)	POS
#55	*Pseudomonas aeruginosa*	20/11/2018	IMP1	POS (7.78 min)	POS

Table S5. Nucleotide sequences for synthetic double-stranded DNA containing *mcr*-1 to *mcr*-9 targets.

gBlock ID	Sequence (5'-3')
KP347127.1_mcr-1.1	ATGATGCAGCATACTTCTGTGTGGTACCGACGCTCGGTCAGTCCGTTTGTTCTTGTGGC<br>GAGTGTTGCCGTTTTCTTGACCGCGACCGCCAATCTTACCTTTTTTGATAAAATCAGCC<br>AAACCTATCCCATCGCGGACAATCTCGGCTTTGTGCTGACGATCGCTGTCGTGCTCTTT<br>GGCGCGATGCTACTGATCACCACGCTGTTATCATCGTATCGCTATGTGCTAAAGCCTGT<br>GTTGATTTTGCTATTAATCATGGGCGCGGTGACCAGTTATTTACTGACACTTATGGCAC<br>GGTCTATGATACGACCATGCTCCAAAATGCCCTACAGACCGACCAAGCCGAGACCAAG<br>GATCTATTAAACGCAGCGTTTATCATGCGTATCATTGGTTTGGGTGTGCTACCAAGTTT<br>GCTTGTGGCTTTTGTTAAGGTGGATTATCCGACTTGGGGCAAGGGTTTGATGCGCCGA<br>TTGGGCTTGATCGTGGCAAGTCTTGCGCTGATTTTACTGCCTGTGGTGGCGTTCAGCA<br>GTCATTATGCCAGTTTCTTTCGCGTGCATAAGCCGCTGCGTAGCTATGTCAATCCGATC<br>ATGCCAATCTACTCGGTGGGTAAGCTTGCCAGTATTGAGTATAAAAAAGCCAGTGCGC<br>CAAAAGATACCATTTATCACGCCAAAGACGCGGTACAAGCAACCAAGCCTGATATGCGT<br>AAGCCACGCCTAGTGGTGTTCGTCGTCGGTGAGACGGCACGCGCCGATCATGTCAGC<br>TTCAATGGCTATGAGCGCGATACTTTCCCACAGCTTGCCAAGATCGATGGCGTGACCA<br>ATTTTAGCAATGTCACATCGTGCGGCACATCGACGGCGTATTCTGTGCCGTGTATGTTC<br>AGCTATCTGGGCGCGGATGAGTATGATGTCGATACCGCCAAATACCAAGAAAATGTGC<br>TGGATACGCTGGATCGCTTGGGCGTAAGTATCTTGTGGCGTGATAATAATTCGGACTCA<br>AAAGGCGTGATGGATAAGCTGCCAAAAGCGCAATTTGCCGATTATAAATCCGCGACCA<br>ACAACGCCATCTGCAACACCAATCCTTATAACGAATGCCGCGATGTCGGTATGCTCGTT<br>GGCTTAGATGACTTTGTCGCTGCCAATAACGGCAAAGATATGCTGATCATGCTGCACCA<br>AATGGGCAATCACGGGCCTGCGTATTTTAAGCGATATGATGAAAAGTTTGCCAAATTCA<br>CGCCAGTGTGTGAAGGTAATGAGCTTGCCAAGTGCGAACATCAGTCCTTGATCAATGC<br>TTATGACAATGCCTTGCTTGCCACCGATGATTTCATCGCTCAAAGTATCCAGTGGCTGC<br>AGACGCACAGCAATGCCTATGATGTCTCAATGCTGTATGTCAGCGATCATGGCGAAAG<br>TCTGGGTGAGAACGGTGTCTATCTACATGGTATGCCAAATGCCTTTGCACCAAAAGAAC<br>AGCGCAGTGTGCCTGCATTTTTCTGGACGGATAAGCAAACTGGCATCACGCCAATGGC<br>AACCGATACCGTCCTGACCCATGACGCGATCACGCCGACATTATTAAAGCTGTTTGATG<br>TCACCGCGGACAAAGTCAAAGACCGCACCGCATTCATCCGCTGA
LT598652.1_mcr-2.1	ATGACATCACATCACTCTTGGTATCGCTATTCTATCAATCCTTTTGTGCTGATGGGTTTG<br>GTGGCGTTATTTTTGGCAGCGACAGCGAACCTGACATTTTTTGAAAAAGCGATGGCGG<br>TCTATCCTGTATCGGATAACTTAGGCTTTATCATCTCAATGGCGGTGGCGGTGATGGGT<br>GCTATGCTACTGATTGTCGTGCTGTTATCCTATCGCTATGTGCTAAAGCCTGTCCTGATT<br>TTGCTACTGATTATGGGTGCGGTGACGAGCTATTTTACCGATACTTATGGCACGGTCTA<br>TGACACCACCATGCTCCAAAATGCCATGCAAACCGACCAAGCCGAGTCTAAGGACTTG<br>ATGAATTTGGCGTTTTTTGTGCGAATTATCGGGCTTGGCGTGTTGCCAAGTGTGTTGGT<br>CGCAGTTGCCAAAGTCAATTATCCAACATGGGGCAAAGGTCTGATTCAGCGTGCGATG<br>ACATGGGGTGTCAGCCTTGTGCTGTTGCTTGTGCCGATTGGACTATTTAGCAGTCAGTA<br>TGCGAGTTTCTTTCGGGTGCATAAGCCAGTGCGTTTTTATATCAACCCGATTACGCCGA<br>TTTATTCGGTGGGTAAGCTTGCCAGTATCGAGTACAAAAAAGCCACTGCGCCAACAGA<br>CACCATCTATCATGCCAAAGACGCCGTGCAGACCACCAAGCCGAGCGAGCGTAAGCC<br>ACGCCTAGTGGTGTTCGTCGTCGGTGAGACGGCGCGTGCTGACCATGTGCAGTTCAAT<br>GGCTATGGCCGTGAGACTTTCCCGCAGCTTGCCAAAGTTGATGGCTTGGCGAATTTTA<br>GCCAAGTGACATCGTGTGGCACATCGACGGCGTATTCTGTGCCGTGTATGTTCAGCTA<br>TTTGGGTCAAGATGACTATGATGTCGATACCGCCAAATACCAAGAAAATGTGCTAGATA<br>CGCTTGACCGCTTGGGTGTGGGTATCTTGTGGCGTGATAATAATTCAGACTCAAAAGG<br>CGTGATGGATAAGCTACCTGCCACGCAGTATTTTGATTATAAATCAGCAACCAACAATA<br>CCATCTGTAACACCAATCCCTATAACGAATGCCGTGATGTCGGTATGCTTGTCGGGCTA<br>GATGACTATGTCAGCGCCAATAATGGCAAAGATATGCTCATCATGCTACACCAAATGGG<br>CAATCATGGGCCGGCGTACTTTAAGCGTTATGATGAGCAATTTGCCAAATTCACCCCCG<br>TGTGCGAAGGCAACGAGCTTGCCAAATGCGAACACCAATCACTCATCAATGCCTATGA<br>CAATGCGCTACTTGCGACTGATGATTTTATCGCCAAAAGCATCGATTGGCTAAAAACGC<br>ATGAAGCGAACTACGATGTCGCCATGCTCTATGTCAGTGACCACGGCGAGAGCTTGGG<br>CGAAAATGGTGTCTATCTGCATGGTATGCCAAATGCCTTTGCACCAAAAGAACAGCGAG<br>CTGTGCCTGCGTTTTTTTGGTCAAATAATACGACATTCAAGCCAACTGCCAGCGATACT<br>GTGCTGACGCATGATGCGATTACGCCAACACTGCTTAAGCTGTTTGATGTCACAGCGG<br>GCAAGGTCAAAGACCGCGCGGCATTTATCCAGTAA
KY924928.1_mcr-3.1	ATGCCTTCCCTTATAAAAATAAAAATTGTTCCGCTTATGTTCTTTTTGGCACTGTATTTTG<br>CATTTATGCTGAACTGGCGTGGAGTTCTCCATTTTTACGAAATCCTTTACAAATTAGAAG<br>ATTTTAAGTTTGGTTTCGCCATTTCATTACCAATATTGCTTGTTGCAGCGCTTAACTTTGT<br>ATTTGTTCCATTTTCGATACGGTATTTAATAAAGCCTTTTTTTGCACTTCTTATCGCACTT<br>AGTGCAATCGTTAGTTACACAATGATGAAGTATAGAGTCTTGTTTGATCAAAACATGATT<br>CAGAATATTTTTGAAACCAATCAAAATGAGGCGTTAGCATATTTAAGCTTACCAATTATA<br>GTATGGGTTACTATTGCTGGTTTTATCCCTGCCATTTTACTTTTCTTTGTTGAAATTGAAT<br>ATGAGGAAAAATGGTTCAAAGGGATTCTAACTCGTGCCCTATCGATGTTTGCATCACTT<br>ATAGTGATTGCGGTTATTGCAGCACTATACTATCAAGATTATGTGTCAGTGGGGCGCAA<br>CAATTCAAACCTCCAGCGTGAGATTGTTCCAGCCAATTTCGTTAATAGTACCGTTAAATA<br>CGTTTACAATCGTTATCTTGCTGAACCAATCCCATTTACAACTTTAGGTGATGATGCAAA<br>ACGGGATACTAATCAAAGTAAGCCCACGTTGATGTTTCTGGTCGTTGGTGAAACCGCTC<br>GTGGTAAAAATTTCTCGATGAATGGCTATGAGAAAGACACCAATCCATTTACCAGTAAAT<br>CTGGTGGCGTGATCTCCTTTAATGATGTTCGTTCGTGTGGGACTGCAACCGCTGTATCC<br>GTCCCCTGCATGTTCTCCAATATGGGGAGAAAGGAGTTTGATGATAATCGCGCTCGCA<br>ATAGCGAGGGCCTGCTAGATGTGTTGCAAAAAACGGGGATCTCCATTTTTTGGAAGGA<br>GAACGATGGAGGCTGCAAAGGCGTCTGCGACCGAGTACCTAACATCGAAATCGAACCA<br>AAGGATCACCCTAAGTTCTGCGATAAAAACACATGCTATGACGAGGTTGTCCTTCAAGA<br>CCTCGATAGTGAAATTGCTCAAATGAAAGGGGATAAGCTGGTTGGCTTCCACCTGATAG

	GTAGCCATGGCCCAACCTACTACAAGCGCTACCCTGATGCTCATCGTCAGTTCACCCC<br>TGACTGTCCACGCAGTGATATTGAAAACTGCACAGATGAAGAGCTCACCAACACCTATG<br>ACAACACCATCCGCTACACCGATTTCGTGATTGGAGAGATGATTGCCAAGTTGAAAACC<br>TACGAAGATAAGTACAACACCGCGTTGCTCTACGTCTCCGATCATGGTGAATCACTGG<br>GAGCATTAGGGCTTTACCTACACGGTACACCGTACCAGTTTGCACCGGATGATCAGAC<br>CCGTGTTCCTATGCAGGTGTGGATGTCACCTGGATTTACCAAAGAGAAAGGCGTTGAT<br>ATGGCGTGTTTGCAGCAGAAAGCCGCTGATACTCGTTACTCACACGATAATATTTTCTC<br>ATCTGTATTGGGTATCTGGGACGTCAAAACATCAGTTTACGAAAAGGGTCTAGATATTTT<br>CAGTCAATGTCGTAATGTTCAATAA
MF543359.1_mcr-4.1	GTGATTTCTAGATTTAAGACGTTATCGGTTAACCAATTCACTTTCATCACTGCGTTGTTTT<br>ATGTTGCCATTTTCAATCTACCGCTCTTTGGTATAGTGCGAAAAGGAATTGAAAAACAAC<br>CAGAAGTTGATCCCCTTTTCATCGCATCTATGCCGCTATTTTTAACATTTGCGCTGAGTT<br>TTTTGTTTTCAATTTTTACCGTCAAATACCTGCTGAAGCCCTTTTTTATCGTATTGACGTT<br>ACTTTCCTCAAGTGTATTTTTTGCAGCCTATCAATACAATGTCGTGTTTGACTACGGCAT<br>GATAGAAAACACGTTTCAAACACATCCTGCTGAAGCATTGATGTATGTAAATCTTGCATC<br>AATTACCAATCTACTGCTGACTGGGCTATTACCGTCATATCTTATTTATAAGGCCGATAT<br>TCATTATCAGCCCTTTTTTAAGGAGTTATTGCATAAATTAGCCTTTATGCTGCTAATGTTC<br>GTTGGCATTGGGATAGTCGCCTTTTTTTACTATCAAGATTATGCTGCATTTGTTCGAAAC<br>AACAGTGAGTTAAGGCGTTACATTGTCCCTACCTATTTTGTCAGTAGTGCATCTAAATAT<br>CTCAATGAGCACTATTTGCAGACGCCCATGGAATACCAACAACTTGGCCTAGATGCGAA<br>GAATGCCAGTCGTAACCCGAACACTAAACCTAACTTATTAGTGGTTGTTGTGGGTGAAA<br>CTGCGCGCTCAATGAGCTATCAATATTATGGATATAACAAGCCAACCAATGCTCATACC<br>CAAAATCAGGGGCTGATTGCGTTTAACGATACTAGCTCATGCGGCACGGCCACGGCGG<br>TGTCTCTACCCTGTATGTTTTCACGAATGGGGCGGGCAGACTATGATCCTCGCCGTGC<br>TAATGCTCAAGACACAGTGATTGATGTGTTAAGTCATAGTGGTATAAAAGTACAGTGGT<br>TTGATAATGATTCTGGCTGTAAAGGTGTGTGTGATCAGGTTGAAAATCTCACGATAGAT<br>TTGAAGAGTGATCCGAAGCTGTGTTCTGGCCAATATTGTTTTGACCAAGTATTGCTCAA<br>CAAATTAGATAAAATTCTGGCAGTAGCACCAAGTCAAGATACAGTAATTTTTTTGCATAT<br>CATTGGTAGTCATGGACCAACTTATTATCTTAGATACCCGCCAGAGCATCGTAAATTTAT<br>ACCGGATTGTCCGCGCAGTGATATTCAAAATTGCAGTCAAGAAGAACTGATTAACACCT<br>ACGACAACACTATTCTATATACGGATTTTATTCTCAGTGAAGTGGTGAATAAATTAAAAG<br>GTAAGCAGGATATGTTCGATACTGCAATGCTGTATCTCTCTGACCATGGTGAGTCTTTG<br>GGTGAAAAGGGCATGTATTTACATGGTGCGCCCTATAGTATTGCACCGAAAGAACAAAC<br>TAGCGTACCAATGCTGGCTTGGGTATCTAATGACTTTAGCCAAGATAATCAGTTGAACA<br>TGACTTGTGTTGCACAGCGAGCAGAACAGGGCGGCTTTTCCCACGACAATTTGTTCGA<br>CAGTTTGCTAGGACTTATGAATGTAAAAACCACCGTCTATCAGAGCCAACTCGATATTTT<br>TGCACCTTGCAGGTATTAG
KY807921.1_mcr-5.1	ATGCGGTTGTCTGCATTTATCACTTTCTTGAAAATGCGCCCGCAAGTGCGCACTGAATT<br>TTTGACTCTGTTCATCAGCCTTGTGTTCACCCTGCTGTGCAATGGCGTGTTTTGGAATG<br>CCCTTCTTGCTGGACGCGACTCCCTAACTTCTGGAACATGGCTAATGCTCCTTTGCACT<br>GGGTTGCTGATCACCGGGCTGCAATGGTTGTTGCTCCTTCTGGTGGCCACGCGCTGGA<br>GTGTCAAGCCACTACTGATTCTGCTTGCTGTCATGACGCCCGCCGCCGTTTATTTCATG<br>CGCAACTACGGGGTTTATCTCGACAAGGCCATGCTGCGGAATCTGATGGAGACGGAC<br>GTCAGGGAAGCCAGTGAGCTGTTGCAATGGAGAATGCTGCCCTACTTGTTGGTTGCAG<br>CCGTATCCGTGTGGTGGATTGCGAGAGTCAGGGTTTTACGAACGGGCTGGAAACAAGC<br>GGTAATGATGCGCAGCGCTTGTCTGGCTGGCGCTCTCGCCATGATTTCCATGGGTCTG<br>TGGCCAGTCATGGATGTGCTGATACCCACGCTTCGTGAAAACAAGCCGCTTCGCTATTT<br>GATCACTCCTGCAAACTACGTCATCTCGGGCATTCGGGTTTTGACTGAACAGGCGTCAT<br>CGTCAGCAGACGAAGCAAGGGAAGTCGTTGCAGCCGATGCGCATCGAGGGCCTCAAG<br>AACAAGGCCGCCGTCCTCGTGCTCTCGTACTGGTTGTCGGGGAAACCGTCAGGGCGG<br>CTAATTGGGGGTTGAGCGGCTATGAACGACAAACCACCCCTGAGTTGGCCGCACGCG<br>ACGTGATCAATTTTTCCGATGTCACCAGTTGCGGGACGGATACGGCTACATCCCTTCCC<br>TGCATGTTTTCCCTCAATGGTCGGCGCGACTACGACGAACGCCAGATTCGTCGGCGCG<br>AGTCCGTGCTGCACGTTTTAAACCGTAGTGACGTCAACATTCTCTGGCGCGATAACCA<br>GTCGGGCTGTAAAGGCGTCTGTGATGGACTGCCCTTTGAAAACCTGTCTTCGGCAGGC<br>CATCCCACACTGTGCCATGGCGAGCGCTGCCTGGATGAAATTCTGCTCGAAGGGTTGG<br>CCGAGAAGATAACAACAAGCCGCAGCGATATGCTGATCGTTCTGCATATGCTGGGCAA<br>TCACGGCCCAGCGTATTTCCAGCGCTATCCCGCAAGCTACCGACGCTGGTCGCCAACC<br>TGCGACACCACCGATCTGGCCAGCTGTTCGCATGAAGCCTTGGTGAACACCTACGACA<br>ACGCCGTGCTTTACACCGATCATGTGCTTGCCCGTACCATTGACCTGCTGTCCGGCAT<br>CCGCTCACACGACACGGCGCTGCTGTACGTTTCCGATCATGGGGAATCGCTCGGCGA<br>GAAAGGCCTGTATCTCCATGGCATACCTTACGTCATCGCGCCGGATGAGCAGATCAAG<br>GTGCCGATGATCTGGTGGCAGTCGAGTCAGGTTTATGCCGACCAAGCCTGTATGCAAA<br>CTCATGCCTCTCGGGCACCGGTAAGTCACGATCACCTGTTTCACACCTTGCTCGGGAT<br>GTTCGACGTGAAAACCGCTGCCTACACGCCAGAGTTGGACCTTCTGGCAACATGCAGA<br>AAAGGACAACCACAATGA
MF176240.1_mcr-6.1	ATGACACAGCATAGTCCTTGGTACCGCCGTCCGGTCAATCCCTATCTGTTGATGAGCG<br>TGGTCGCTTTATTTTTGTCAGCGACAGCAAACCTAACTTTCTTTGATAAAATCACCAATA<br>CTTATCCGATGGCACAAAACGCAGGCTTTGTGATCTCAACGGCGCTTGTGCTATTTGG<br>GGCGATGCTATTGATTACTGTGCTGTTATCGTATCGCTATGTGCTTAAGCCTGTGTTGA<br>TTTTGCTGCTTATCATGGGTGCGGTGACGAGCTATTTTACCGATACTTATGGCACCGTT<br>TATGACACCACCATGCTCCAAAATGCCTTGCAAACTGACCAAGCCGAGTCTAAGGACTT<br>GATGAATATGGCGTTTTTTGTGCGGATTATCGGGCTTGGCGTGTTGCCAAGTATCTTGG<br>TGGCGTGGGTCAAGGTGGATTATCCGACATTGGGTAAGAGTCTGATTCAGCGTGCGAT<br>GACTTGGGGTGTGGCAGTGGTGATGGCACTTGTGCCGATTTTGGCATTTAGTAGTCAC<br>TACGCCAGTTTCTTTCGTGAACATAAGCCACTGCGTAGCTATGTCAATCCCGTGATGCC<br>GATTTATTCAGTAGGTAAGCTTGCCAGTATTGAGTACAAAAAAGCCACCGCGCCAAAAG<br>ACACCATCTATCATGCCAAAGATGCTGTACAGACGACGACGCCTGCCGAGCGTAAGCC<br>ACGACTCGTGGTGTTCGTCGTCGGTGAGACGGCTCGAGCTGACCATGTGCAGTTTAAT<br>GGCTATAGTCGTGAGACTTTTCCGCAGCTTGCCAAGATTGACAACCTAGCCAATTTTAG<br>CCAAGTGACATCGTGTGGCACATCGACGGCGTACTCTGTGCCGTGTATGTTCAGTTAT

	CTGGGTCAAGATGACTATGATGTCGATACCGCCAAATACCAAGAAAACGTGCTGGATA<br>CGCTTGACCGACTGGGTGTGGGTATCCTGTGGCGGGATAATAATTCAGACTCAAAAGG<br>CGTGATGGATAAACTGCCTGCTTCGCAGTATTTTGATTATAAATCAGCGACCAACAACA<br>CCATCTGTAACACCAATCCTTACAACGAATGTCGTGATGTCGGTATGTTGGTGGGGCTA<br>GATGATTATGTGAGTACCAATCAAGGCAAAGATATGCTCATCATGCTACACCAAATGGG<br>TAATCATGGGCCGGCGTACTTCAAGCGTTATGACGAGCAATTTGCCAAATACACCCCTG<br>TGTGCGAAGGTAATGAACTTGCCAAGTGTGAACACCAATCGCTCATCAACGCCTATGAT<br>AATGCACTGCTTGCGACCGATGATTTTATCGCCAAAAGTATCGATTGGCTAAAAACGCA<br>TCAGGCCAACTATGATGTTGCCATGCTCTATGTCAGCGACCACGGCGAGAGTCTGGGT<br>GAAAATGGCGTCTATCTGCATGGTATGCCAAATGCCTTTGCACCAAAAGAACAGCGAG<br>CGGTACCGGCATTCTTTTGGTCAAATAATCCATCGTTCACGCCAACTGCCAGCGACACT<br>GTGCTGACACATGATGCGATTACGCCGACTCTACTGAAGCTGTTTGATGTCACAGCGG<br>ATAAGGTCAAAGACCGCACCGCATTCATCCGCTGA
MG267386.1_mcr-7.1	ATGCGCATCACGCTCGGTGTGATGAAGGTGAATTTGTTGCTGGTGCTCTTTTTCGCACT<br>GGTGCTGAACTGGCCTTTCTTTCTTCGTTTTTATTCTGTTATCAGTGGTCTGGAACATGT<br>CCGGGCCGGTTTCGTTATCTCGGTTCCTCTGGTGCTGCTTGCCGCACTCAACGCCGTC<br>TTTATCCCCTTTACCTTCCGCTGGTTGCTCAAGCCCTTCTTTTCGTTGTTGATCCTGACA<br>GGCTCCATCGTCAGTTACGCCATGCTCAAATACGGCGTCATCTTCGATGCCAGCATGA<br>TCCAGAACATAGTGGAGACCAACAACAGTGAGGCGACCTCCTACCTGAATGTGCCGGT<br>CGTGCTCTGGTTCCTGCTGACCGGTGTGTTGCCCATGGTGGTGCTCTGGTCGCTGAAG<br>GTGCGCTATCCGGCAAACTGGTACAAGGGGCTGGCCATCAGGGCTGGTGCTCTGGCC<br>TTCTCGCTGCTGTTCGTGGGAGGCGTTGCCGCACTTTACTATCAGGATTACGTCTCGAT<br>CGGCCGCAATCACCGGATCCTGGGCAAGCAGATAGTGCCGGCCAACTATGTCAACGG<br>CATCTACAAATATGCCCGCGACGTGGTATTTGCTACCCCCATCCCTTATCAACCGCTGG<br>GGACTGATGCCAAAGTCGTCGCCAAAGGGGATAAACCGACCCTGATGTTTCTGGTGGT<br>GGGGGAGACAGCCCGCGGCAAGAACTTCTCGATGAACGGCTACGAGAAAGAGACCAA<br>CCCCTTTACCAGTCAGGCCGGGGGCGTGATCTCCTTCAAGGACGTGCGCTCTTGCGG<br>CACGGCCACAGCGGTGTCGGTGCCCTGCATGTTCTCCAACATGGGGCGCAAGGAGTT<br>TGATGACAACCGGGCCCGCAACAGCGAAGGCCTGCTCGATGTGCTGCAAAGAAGCGG<br>GGTCTCCATCTTCTGGAAGGAGAACGACGGCGGCTGCAAAGGGGTGTGCGATCGGGT<br>GCCCAACATCGAGATCAAGCCAAAAGATCACCCACAGTTCTGCGACAAGAACACCTGC<br>TATGACGAGGTTGTACTGCAAAATCTCGACGACGAGGTGGCGCAGATGAAGGGCGAC<br>AAGCTGGTCGGTTTCCATCTGATCGGCAGCCACCGCCCGCCCTACCACCAACGCTATC<br>CGGACAAACCACCCCCGTTCGTACCGGACTGCCCGCGCAGCGACATCGAGAACTGCA<br>GCGATGAAGAGCTGGTCAACACCTATGACAACACCATCCGCTACACCGATTTTGTCATA<br>GCAGAGATGATTACCAAGCTGAAAAAGTATGAAGATAAGTACAACACGGCGTTGATCTA<br>CCTCTCTGATCACGGCGAGTCGCTGGGTGCGATGGGGCTCTATCTGCATGGCACGCC<br>CTACAAGTTTGCCCCTGACGACCAGACCCGGGTACCGATGCAGGTCTGGATGTCGCC<br>GGGCTTTGCCAAAGAGAAGGGGATGGATCTGAACTGCCTGCAGCAAAAAGCGGCAGA<br>CAATCGCTACTCCCATGACAACCTCTTCTCCTCTGTGCTCGGGATCTGGGATGTCAGCA<br>CGGCGGTGTACGACAAGCAGCTCGATATTTTCAGCCAGTGCCGCACCGTGCAGTAA
NG061399.1_mcr-8.1	TAATCCTTGGAAACCTTAGAAATTGATGGAGGATCTTAACAAGATCCTGACATAGATTTT<br>CAGATACTGCGTACGATTTGTTAATCTTCAGGAATCGTGCATGTTCAAGTATCTTTTATC<br>TTTCAAACTGAACCCGGTACAACGGACCTGGGCTGCAGCATTTTTTTTCACTACAATCG<br>GCAACATAGCACTTTGGCAAACACTATGGATTAATGTAGATGTTCATAATATACATAATC<br>TACTTTTTTTTGCCAGTCTGCCAATATTTCTTTTCTGCTTTCTAAGTATCTTACTTACACC<br>AGTCATGGTTATTCCATATTTATGCAGGCCTCTACTTGTAGTTCTTATTCTAATCAGTGC<br>CTGCTGTAGTTATTTCATGATGAAATACAACATATTAATTGACCGCAGCATGGTGCAAAA<br>CTTTTTTGAGACTAATCAGGCTGAATTAACATCATACTTATCCGTTCCTTTTCTTTCCACT<br>CTATTTCTACTTGGCATTGTACCAGCAATTATCCTGGCGTTGCCTTCAACAGACAATAAG<br>CGGGGAGCTTTTAGAATTGAATTGTGGTGGTTGGCGCATATTTGCATAGCTGTAGTCTT<br>ATTAGCCATGGTTACCATGGTGTTTTATAAGGATTACGCATCTCTCATACGAAACAATAT<br>GCAGATTAAAGACCAGGCTTTACCTTTTAACTTTGTGCGTAATACGAATGGTTACCTTAA<br>AAGAAAATACCAGGCATCTTCAACAATTCTACAAAGCGTGGGGGAGGATGCTGTACGT<br>CCAATATATTCAAATGCTCCACCGAAACTGGTGGTTGTCGTCGTGGGCGAAACCGCCA<br>GAGCACAGAATTTCCAGCTGAATGGCTATTCGCGGGTAACCAACCCCTATCTTTCCAGA<br>CGACATGATGTTATCAGTTTCAAAAATGTGTCGTCATGCGGAACGGCTACCGCAATATC<br>ACTACCCTGCATGTTCTCGCGAATGTCACGTAACGAATACAATGAAGTCCGTGCCGCAT<br>CAGAAGAAAACTTGCTGGATATCCTTAAACGTACAGGTGTTGAGGTGCTATGGCGCAA<br>CAATAACAATGGTGGTTGTAAGGGAATCTGCAAGCGAGTACCCACAGATGATATGCCG<br>GCAATGAAAGTAATTGGGGAATGTGTTAACAAAGATGGTACATGCTTTGATGAGGTGTT<br>ATTAAATCAACTCTCATCCCGAATTAATGCAATGCAGGGTGATGCGCTTATTGTTTTACA<br>TCAAATGGGCAGTCATGGACCAACATATTTTGAACGTTATCCGTCTACAAGTAAAGTCTT<br>TAGCCCAACTTGCGACAGCAACCTGATCGAAAAATGCTCAAATAAAGAACTGGTCAATA<br>CATACGACAATACGCTAGTTTATACTGATCGTATGCTGAGCAAAACTATTGAACTGTTGC<br>AACGTTATTCCGGGATGCGTGACGTTGCTATGATATATCTTTCTGATCATGGAGAATCG<br>CTGGGGGAAAGCGGAATATATCTTCATGGCACACCATATATTATTGCCCCCAATGAACA<br>AACACACATCCCGATGTTTATGTGGTTTTCGTCTTCATTCGCGCAGCATTCCAAATTAAA<br>TCTAGAATGCCTGACCGGTAATGCCGACAAACAATACAGTCATGATAATTTTTATCATTC<br>AATACTTGGTCTCTTCAACGTAAAAACCAGTGTATATAAACCGGAGTTAGATATGTTTAC<br>TCTATGTCGACAATCTGACCACACACCACTGTCTTCCGCAGTTGTAAGAGAGAAAACAG<br>ATGGGAATGGTTAGTAAATAAACTATCATCTTATTTATTTCACCACCCTATAACGCACTA<br>TACAACACCTGCTCCTTAAATATAAACAAGGAGCAGTCATTGACAATTTATGGC
NG_064792.1_mcr-9.1	ATGCCTGTACTTTTCAGGGTGAAAGTTATTCCGCTGGTTTTACTTCTGGCAATGATCTTT<br>GCGTTTTTACTTAACTGGCCAATATTGCTGCATTTTTACGAGATTTTGTCGCATTTAGAG<br>CATGTCAAAATTGGTTTTGTCATTTCTATTCCCTTTGTTCTGGTTGCGGCGCTTAACGTT<br>GTTTTTATGCCTTTCTCAGTTCGTTTTCTGCTGAAACCTTTCTTTGCTTTACTGTTTATCA<br>CTGGCTCACTGGTCAGTTATTCGACACTAAAATATAAAGTAATGTTTGATCAAACGATGA<br>TTCAAAACATTATTGAAACTAACCCCCAGGAAGCGCATTCCTATCTTAATGGCTCAATTA<br>TTATATGGTTCGTCTTTACCGGTATCCTTCCTGCCATCCTCCTTTTTTCAATAAAAATTCA<br>ATATCCTGAAAAATGGTATAAAGGCATTGCTTACCGTTTGCTCTCCGTGCTGGCATCGT

	TGAGTTTGATTGCAGGTGTTGCCGCACTTTATTATCAGGATTATGCCTCTGTCGGCCGC<br>AATAACTCGACATTGAATAAAGAGATCATCCCGGCGAACTACGCTTACAGCACTTTCCA<br>GTATGTTAAGGATACGTACTTTACGACTAAAGTGCCTTTCCAGACGCTGGGGAATGATG<br>CTAAACGCGTCGTCGCTCACGAAAAACCCACGCTGATGTTCCTGGTGATTGGCGAAAC<br>GGCACGCAGCCAGAATTTCTCGATGAACGGTTATTCGCGTGATACCAATGCCTTTACCA<br>GCAAATCCGGCGGCGTTATTTCGTTTAAAAATATGCATTCCTGCGGTACCGCTACCGCA<br>ATATCCGTTCCGTGCATGTTCTCGAATATGAATCGCACCGAGTACGACAGTAAAAAAGC<br>ATCTAACAGTGAAAATTTCCTCGACATCGTGCAGAAAACCGGTGTCTCGCTGTTATGGA<br>AAGAGAACGATGGCGGTTGTAAAGGCGTATGTAGCCGCATCCCGACTGTCGAAATTAA<br>GCCTAGTGATAACCCGAAACTGTGCGATGGCAAAACGTGCCATGACGAGGTGATGCTG<br>GAAAACCTTGATGATGAAATCGCCAAAATGCCAGGTGATAAGCTTGTCGCCTTCCATAT<br>CATTGGCAGCCATGGACCGACTTATTACCTGCGTTATCCGGCTGAGCATCGCCACTTC<br>ATGCCCGAATGTGCACGTAGCGATATCGAAAACTGTACTCAGGAACAATTGGTCAACAC<br>CTACGACAACACCCTTCGTTATACAGACTATGTATTAGCTGAGATGATTGAAAAGCTAAA<br>AAATTACAGCGATCAGTACAACACCGTGCTGCTTTATGTGTCCGATCATGGTGAATCAT<br>TGGGCGAAAGCGGGCTATATCTGCACGGCACGCCGTACAAACTGGCACCGGATCAGC<br>AGACGCATATTCCGATGCAGGTCTGGATGTCACCGGGCTTTATCGCCGGGAAACACAT<br>CAACATGTCTTGCCTTGAAAATAATGCGGCGAAAAAATCATATTCCCACGACAACCTGT<br>TCTCATCGATTTTGGGGCTGTGGGACGTAAGCACCAGCGTCTATAATCCTGACCGCGA<br>TTTGTTCCGCGAATGCCGTGGCTAA

Table S6. Accession numbers list of genomes used in the alignment for LAMP primer design.

Accession numbers (mcr gene variants)		
KP347127.1 (mcr-1.1)	LT598652.1 (mcr-2.1)	MG564491.1 (mcr-3.12)
KX236309.1 (mcr-1.2)	MF176239.1 (mcr-2.2)	MG822663.1 (mcr-4.2)
KU934208.1 (mcr-1.3)	KY924928.1 (mcr-3.1)	MG822665.1 (mcr-4.4)
KY041856.1 (mcr-1.4)	NMWW01000143.1 (mcr-3.2)	MG822664.1 (mcr-4.5)
KY283125.1 (mcr-1.5)	MF495680.1 (mcr-3.3)	MF543359.1 (mcr-4.1)
KY352406.1 (mcr-1.6)	FLXA01000011.1 (mcr-3.4)	MG026621.1 (mcr-4.3)
KY488488.1 (mcr-1.7)	MF489760.1 (mcr-3.5)	KY807921.1 (mcr-5.1)
KY683842.1 (mcr-1.8)	MF598076.1 (mcr-3.6)	MG384740.1 (mcr-5.2)
KY964067.1 (mcr-1.9)	MF598077.1 (mcr-3.7)	MF176240.1 (mcr-6.1)
MF176238.1 (mcr-1.10)	MF598078.1 (mcr-3.8)	MG267386.1 (mcr-7.1)
KY853650.2 (mcr-1.11)	MF598080.1 (mcr-3.9)	MG736312.1 (mcr-8.1)
LC337668.1 (mcr-1.12)	MG214531.1 (mcr-3.10)	NG064792.1 (mcr-9.1)
MG384739.1 (mcr-1.13)	MG489958.1 (mcr-3.11)	MK791138.1 (mcr-9.1)

https://www.ncbi.nlm.nih.gov/genbank/ (last accessed March 29, 2020)

Figure S1. Chip output obtained during amplification for positive isolates.

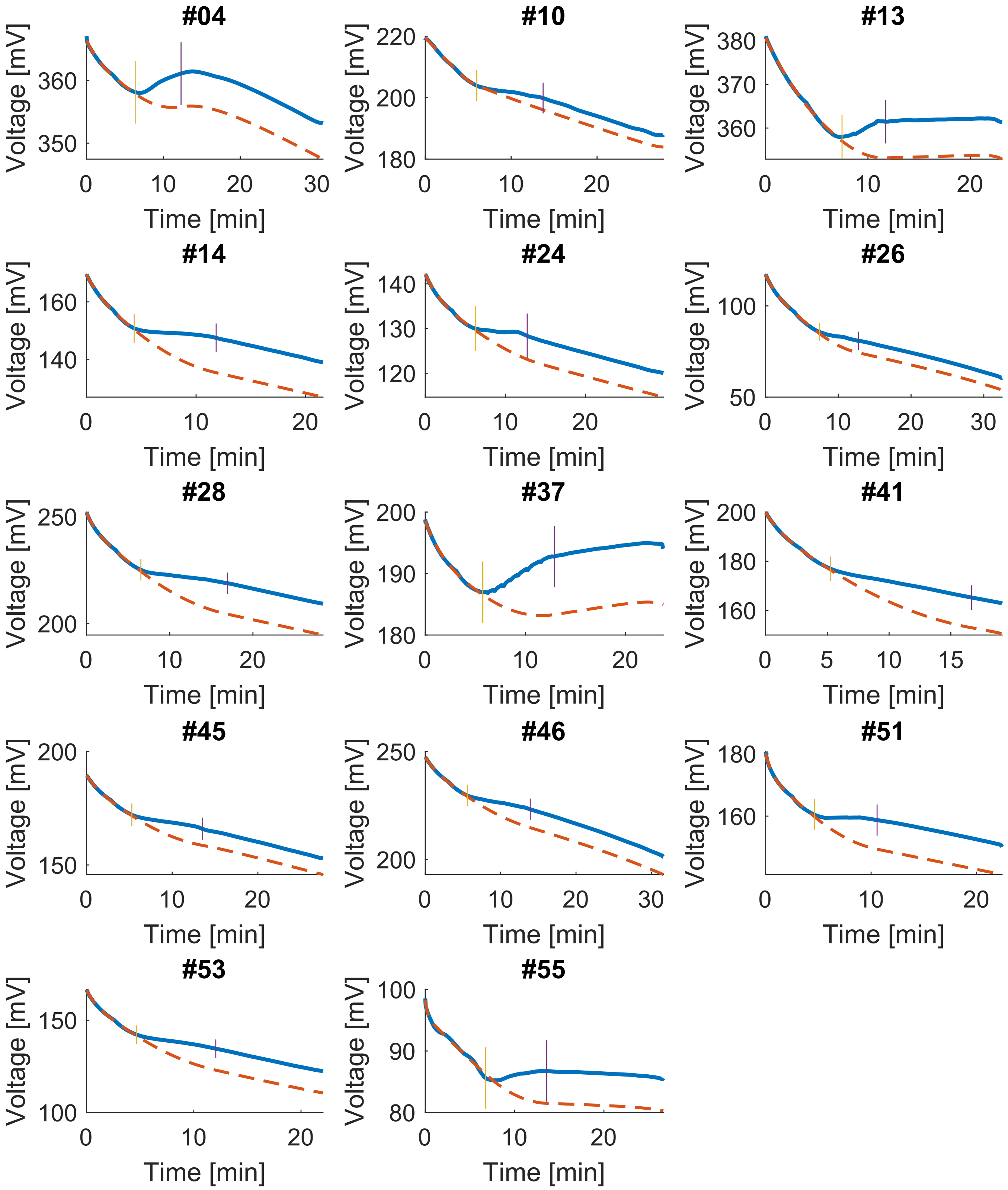

#: Isolate ID.

Figure S2. Drift compensation for positive isolates.

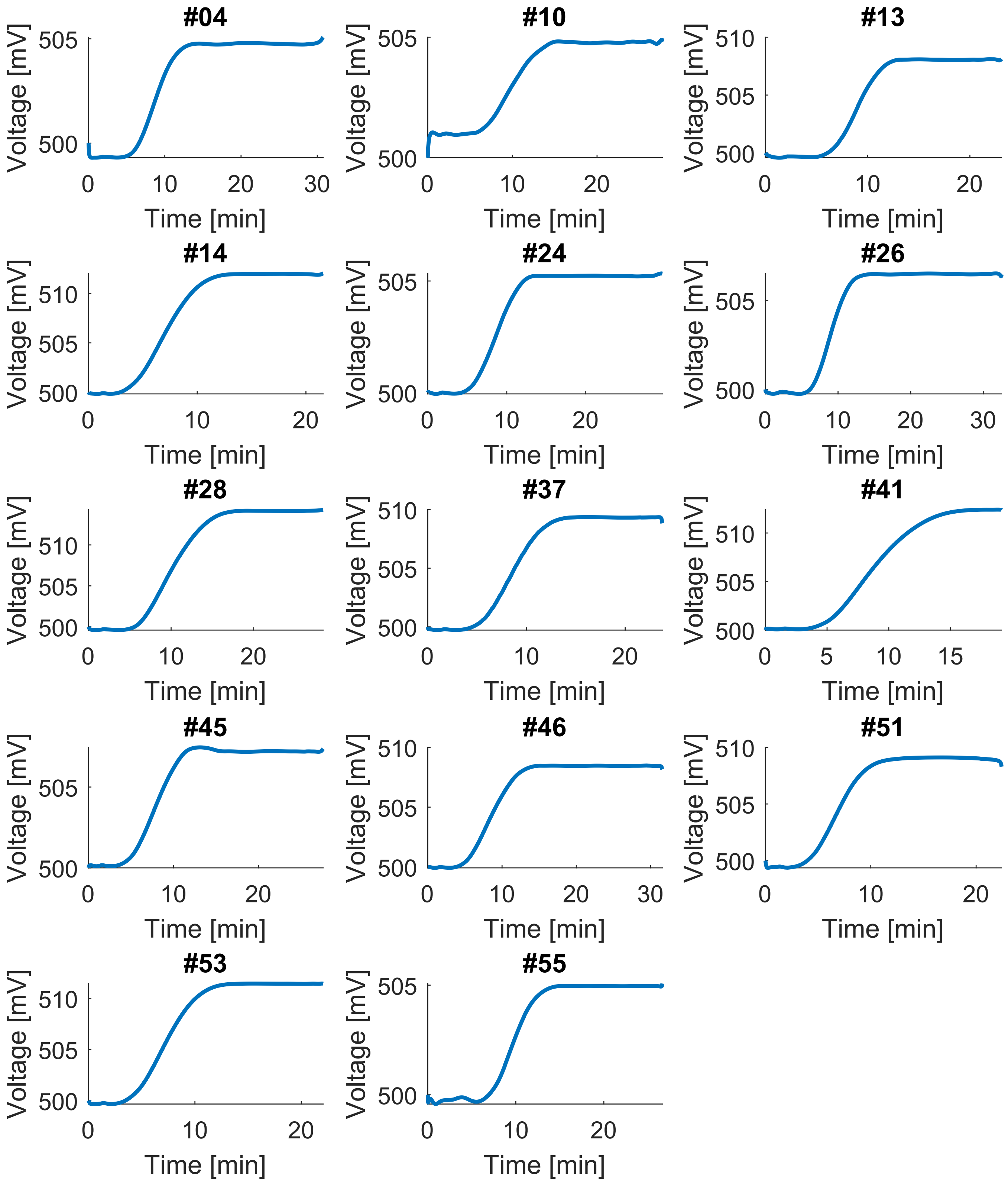

#: Isolate ID.

Figure S3. Linearization, normalization and sigmoidal fitting for positive isolates.

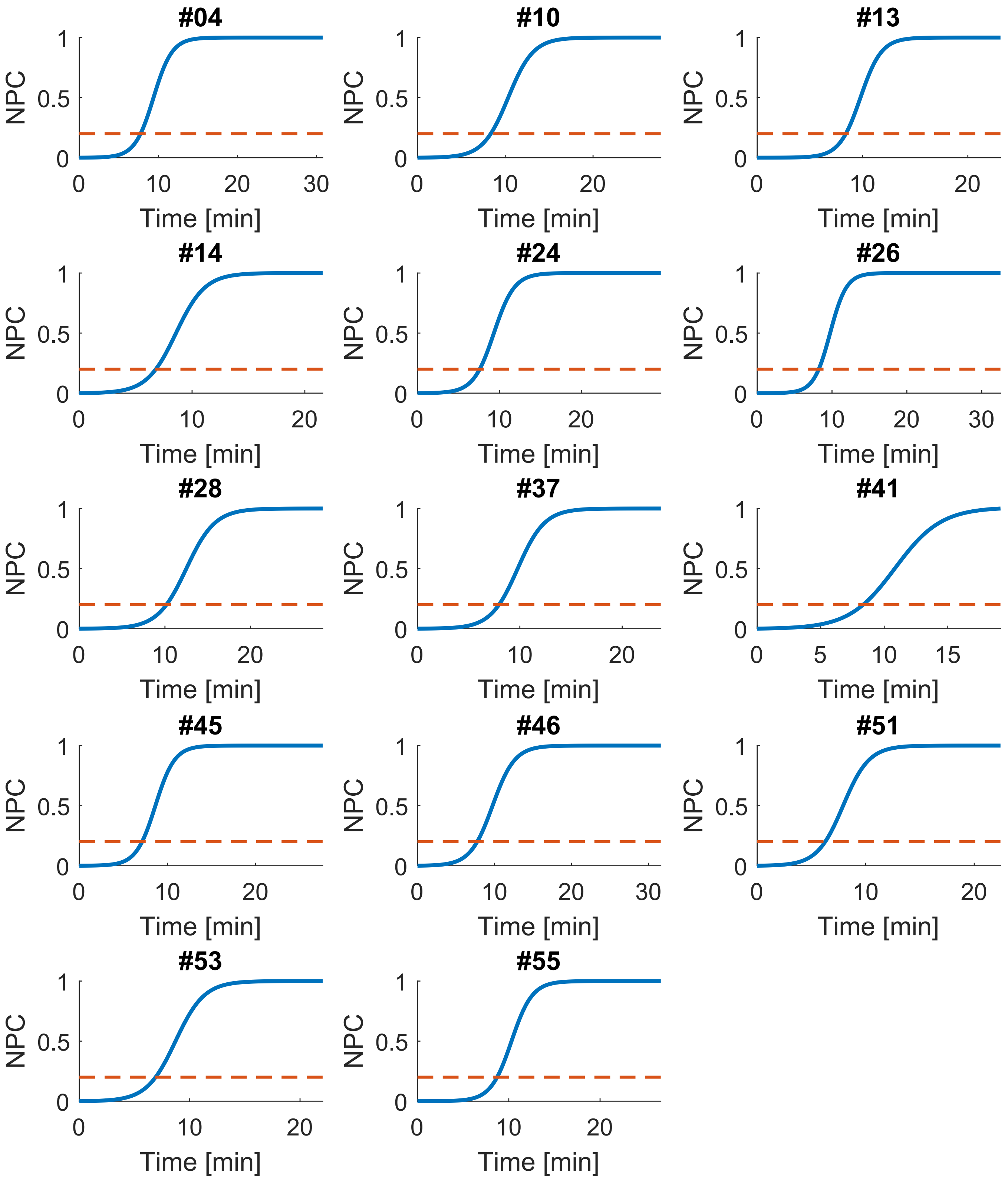

#: Isolate ID.

Figure S4. Data processing for negative isolates.

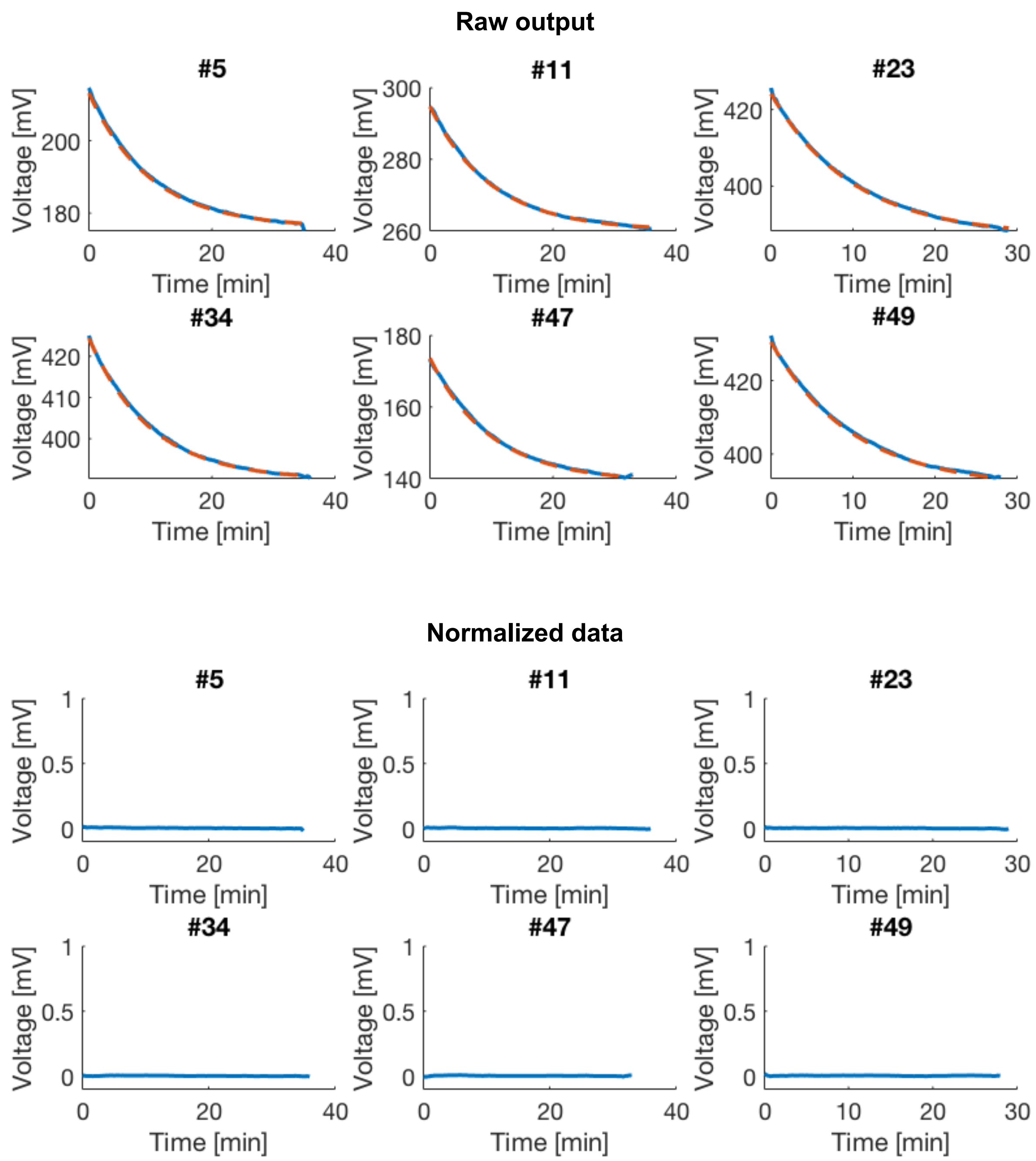

#: Isolate ID.

Figure S5. Annotated photograph of the LoC platform without the case.

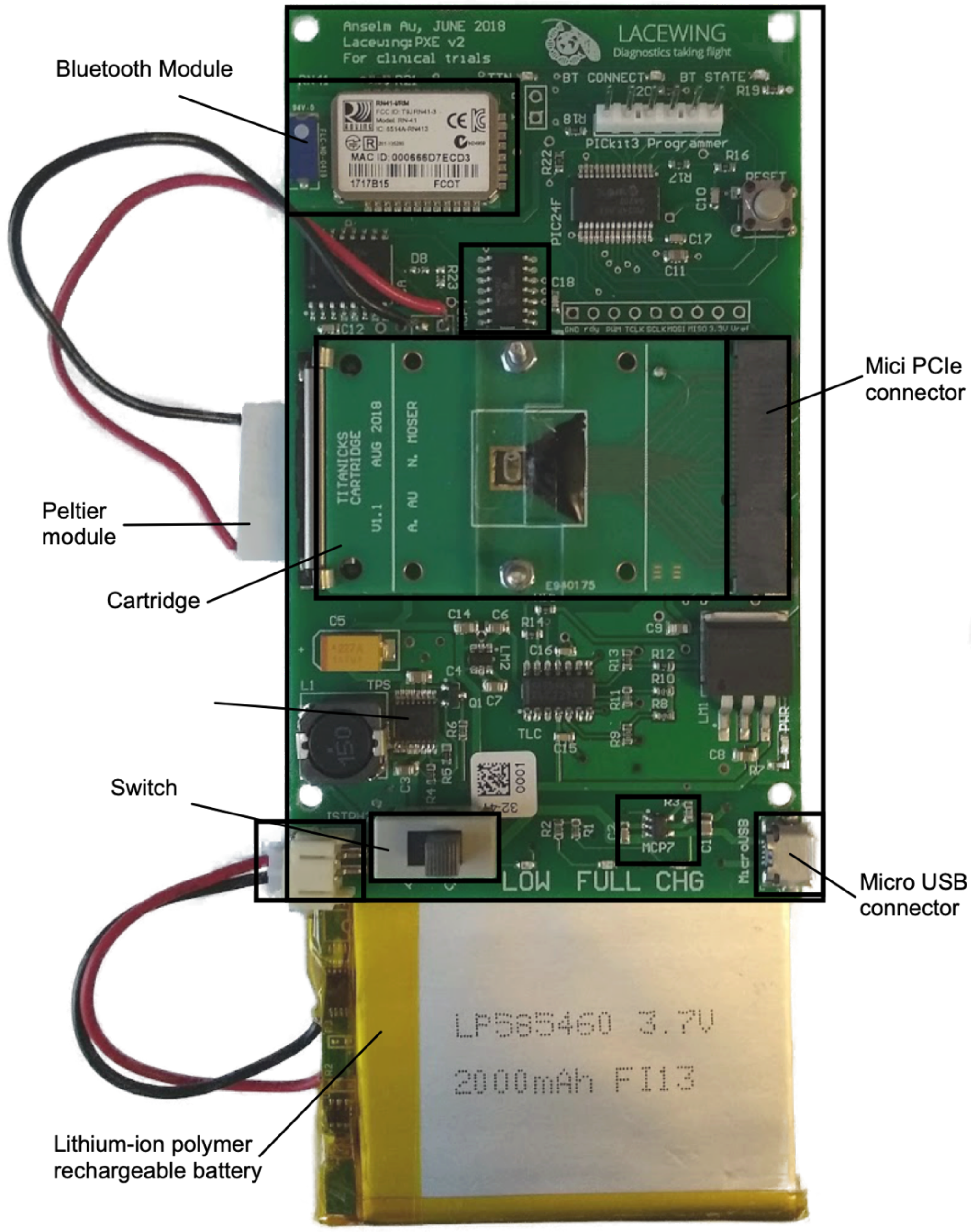

Supplement: Supplementary file 1 — Supplementary information. [file 41598_2020_64612_MOESM1_ESM.pdf]
